# Monocyte (THP-1) Response to Silver Nanoparticles Synthesized with *Rumex hymenosepalus* Root Extract

**DOI:** 10.3390/nano14010106

**Published:** 2024-01-02

**Authors:** Francisco Javier Alvarez-Cirerol, José Manuel Galván-Moroyoqui, Ericka Rodríguez-León, Carmen Candía-Plata, César Rodríguez-Beas, Luis Fernando López-Soto, Blanca Esthela Rodríguez-Vázquez, José Bustos-Arriaga, Adriana Soto-Guzmán, Eduardo Larios-Rodríguez, Juan M. Martínez-Soto, Aaron Martinez-Higuera, Ramón A. Iñiguez-Palomares

**Affiliations:** 1 Departamento de Ciencias Químico Biológicas y de la Salud, Universidad de Sonora, Hermosillo 83000, Mexico; francisco.alvarez@unison.mx; 2 Departamento de Medicina y Ciencias de la Salud, Universidad de Sonora, Hermosillo 83000, Mexico; carmen.candia@unison.mx (C.C.-P.); luisfernando.lopez@unison.mx (L.F.L.-S.); adriana.soto@unison.mx (A.S.-G.); juanmanuel.martinez@unison.mx (J.M.M.-S.); 3 Departamento de Física, Universidad de Sonora, Hermosillo 83000, Mexico; ericka.rodriguez@unison.mx (E.R.-L.); cesar.rodriguez@unison.mx (C.R.-B.); blanca.rodriguezvazquez@unison.mx (B.E.R.-V.); 4 Facultad de Estudios Superiores Iztacala, Universidad Nacional Autónoma de México, Ciudad de México 04510, Mexico; jose.bustos@iztacala.unam.mx; 5 Departamento de Ingeniería Química y Metalurgia, Universidad de Sonora, Hermosillo 83000, Mexico; eduardo.larios@unison.mx; 6 Departamento de Agronomía, Universidad de Sonora, Hermosillo 83000, Mexico; aaron.martinez@unison.mx

**Keywords:** silver nanoparticles, monocytes, *Rumex hymenosepalus*

## Abstract

The study, synthesis, and application of nanomaterials in medicine have grown exponentially in recent years. An example of this is the understanding of how nanomaterials activate or regulate the immune system, particularly macrophages. In this work, nanoparticles were synthesized using *Rumex hymenosepalus* as a reducing agent (AgRhNPs). According to thermogravimetric analysis, the metal content of nanoparticles is 55.5% by weight. The size of the particles ranges from 5–26 nm, with an average of 11 nm, and they possess an fcc crystalline structure. The presence of extract molecules on the nanomaterial was confirmed by UV-Vis and FTIR. It was found by UPLC-qTOF that the most abundant compounds in Rh extract are flavonols, flavones, isoflavones, chalcones, and anthocyanidins. The viability and apoptosis of the THP-1 cell line were evaluated for AgRhNPs, commercial nanoparticles (AgCNPs), and Rh extract. The results indicate a minimal cytotoxic and apoptotic effect at a concentration of 12.5 μg/mL for both nanoparticles and 25 μg/mL for Rh extract. The interaction of the THP-1 cell line and treatments was used to evaluate the polarization of monocyte subsets in conjunction with an evaluation of CCR2, Tie-2, and Arg-1 expression. The AgRhNPs nanoparticles and Rh extract neither exhibited cytotoxicity in the THP-1 monocyte cell line. Additionally, the treatments mentioned above exhibited anti-inflammatory effects by maintaining the classical monocyte phenotype CD14++CD16, reducing pro-inflammatory interleukin IL-6 production, and increasing IL-4 production.

## 1. Introduction

Nanotechnology is concerned with the design, fabrication, and study of the properties of materials, structures, or devices in the size range of 1 to 100 nm [1,2]. At this scale, some materials exhibit physicochemical properties that differ significantly from their bulk counterparts due to confinement effects and the large area-to-volume ratio that characterizes them [3]. Some properties of nanomaterials, such as their versatile plasmonic response, their microbicidal activity, or their great catalytic response, have been very attractive for various technological sectors, including textiles, electronics, automotive, and food, in addition to the cosmetic and biomedical industries [4,5,6,7]. The production of nanomaterials has grown tremendously in recent years, and it is predicted that the production of nanomaterials will exceed 2.3 megatons by 2028 [8]. In the case of metallic nanoparticles (oxides and non-oxides), the global market grew from $22.2 billion in 2022 to $25.76 billion in 2023, representing a growth of 16%, and it is estimated that by 2027 the market growth will be 14% per year [9]. Among the different metallic nanoparticles produced industrially, the production of silver nanoparticles stands out, mainly because of their microbicidal effects. We can find these nanoparticles incorporated in cosmetic products [10,11,12,13], embedded in different matrices such as plastics, natural and synthetic fibers, gels or colloidal dispersions [14,15], food additives [16] and even directly incorporated in food. 

This means that individuals in developed countries are currently exposed to significant doses of these nanomaterials. Regardless of the route of exposure (inhalation, dermal contact, ingestion, or ocular uptake), the immune system, which is the first line of defense against foreign bodies in organisms, will react to the presence of these particles by generating inflammatory processes, a factor of greater incidence in human health [17,18]. For this reason, it is important to know the response of biological systems to exposure to different nanomaterials and also to develop nanoparticles that, in addition to the desired biological effect, have the least adverse impact on human health. In this sense, the “green synthesis” of nanoparticles is an ecological and cost-effective route that exploits the potential of biological materials for the synthesis of metallic nanoparticles. Green synthesis involves the reduction of metal ions using biomass or plant extracts as sources of reducing agents and stabilizers in a one-step synthesis. The nanomaterial obtained consists of a metallic particle surrounded by an organic matrix corresponding to the residual molecules of the extract adsorbed on the metallic surface. These materials have been used in biomedicine for the treatment of diabetes, hypolipidemia, and diseases involving inflammatory processes [19,20,21,22,23,24]. In addition to being cost-effective and environmentally friendly, the biological approach offers several advantages over traditional physical and chemical methods. In particular, the process is highly efficient in catalyzing reactions in aqueous media at standard temperature and pressure. In addition, the process is flexible and can be implemented in almost any environment and at any scale. Biological constituents initiate the reduction, often triggered by various compounds including polyphenols, carbonyls, amines, amide groups, proteins, pigments, flavanones, terpenoids, alkaloids, and other reducing agents [25,26]. The genus *Rumex* L. of the *Polygonaceae* Juss family includes more than 200 species that grow mainly in temperate climates. It has been reported that extracts from different species of *Rumex* present antioxidant, anti-inflammatory, anti-cancer, and antibacterial activity, among others, of clinical interest [27]. *Rumex hymenosepalus* is a species that is native to the southern region of the USA and northern Mexico. Its root is tuber-shaped and contains a high concentration of antioxidant compounds [28]. As a result, extracts from this plant’s root are excellent candidates for the production of metal nanoparticles in the context of green synthesis. The molecules of plant extracts are determined by mass spectroscopy. This allows for the determination of the functional groups present in the extract and the most likely mechanisms for reducing metal precursors and functionalizing metal nanoparticles. When a nanoparticle is introduced into an organism, it encounters monocytes—blood cells that are differentiated by signals triggered by the organism or situation. These cells use surface proteins to perform various activities, and their widely recognized subsets are distinguished by the number of glycoproteins, membrane receptors, displayed on their surface. CD14 and CD16 proteins are used to classify monocytes into subsets based on their expression levels. These subsets include classical monocytes (CD14++CD16−), intermediate CD14+CD16+, and non-classical CD14+CD16++. These proteins help track the response of monocytes in the presence of a foreign agent. The polarization of monocytes leads to the development of a macrophage, which has different effects on the organism. The two main polarized states are M1, which is associated with inflammation and IL-6 expression, and M2, which secretes cytokines such as IL-4 and IL-6 associated with protective and anti-inflammatory functions, including IL10 [29]. In this work, we synthesized silver nanoparticles with *Rumex hymenosepalus* extract and evaluated the immunomodulatory capacity of these particles in THP-1 cells by assessing monocyte polarization through the production of pro-inflammatory (IL-6) and anti-inflammatory (IL-4, IL-10) cytokines. A comparison is made with Rh extract and commercial nanoparticles synthesized by reduction with citrate and stabilized with PVP.

## 2. Materials and Methods

### 2.1. Obtaining Rumex hymenosepalus Extract

Commercially sourced dried slices of *Rumex hymenosepalus* root were macerated in 1L of an ethanol/water mixture (70:30 vol/vol) at room temperature in the dark. A quantity of 150 g of the product was used. Daily measurements of the extract’s absorbance were taken. After ten days, the extraction process was complete, as no significant changes in the absorbance were observed. The resulting liquid was filtered using a vacuum pump-coupled filtration unit with a pore size of 0.2 µm. Half of the filtered extract was reserved for producing silver nanoparticles. Next, the other half of the filtered extract was subjected to a rotary evaporator process to eliminate the ethanol. Then, the aqueous concentrate of the extract was frozen at −80 °C for subsequent lyophilization. The obtained lyophilized powder was stored in vials at 4 °C until use.

### 2.2. Rumex hymenosepalus Sample Preparation for Mass Spectroscopy

To prepare the samples, 100 mg of lyophilized extract was weighed in triplicate, and 2.5 mL of methanol (80% vol) was added. They were mixed in a vortex and centrifuged at 1000× *g* for 25 min at 4 °C. The supernatants were filtered (0.22 µm pore) and placed in a miVac centrifugal concentrator to evaporate the solvent. The pellet was reconstituted in an 80:20 ultrapure water/acetonitrile solution. Samples were kept in amber vials for the study.

### 2.3. Rumex hymenosepalus Metabolites Global Profile by UPLC-MS-QTOF-SYNAPT

A Waters ACQUITY Ultra Performance Liquid Chromatography (UPLC) Class 1, coupled with a quadrupole-time of flight mass spectrometer (Waters Synapt G1 Q-TOF, Markham, ON, Canada) and an electrospray ionization system, was used for compound identification. An ACQUITY CSH C18 column (2.1 mm × 150 mm, 1.7 µm) was used for the chromatographic separation of compounds with a constant flow of 0.2 mL/min and keeping the column temperature constant (40 °C). As mobile phase, 0.1% formic acid in water (A) and acetonitrile (B) were used. Percent A (% A) was used to describe the gradients used for the mobile phase as follows: it started at 90% for 0.5 min, then for 16.5 min, it decreased linearly to 20%. It was held at 20% for 1 min, then rapidly decreased for 0.1 min until reaching 0%. It was maintained for 2 min at 0%, and later, it was increased for 0.1 min up to 90%, keeping it until reaching 25 min. The mass spectrometric data were collected in the negative ion mode. The capillary voltage was 2.3 kV, the sampling cone voltage was 35 V, and the extraction cone voltage was 3.5 The source temperature, desolvation temperature, and desolvation gas flow were 120 °C, 300 °C, and 500 L/h.

### 2.4. Silver Nanoparticle Synthesis and Their Characterization

For the synthesis of nanoparticles, AgNO_3_ solution 0.1 M (Sigma-Aldrich, San Luis, MO, USA) with 99.9% purity and *Rumex hymenosepalus* extract, obtained with modifications to the protocol reported in previous works [20], were used. The extract of *Rumex hymenosepalus*, measuring 60 mL, was mixed with a 0.1 M solution of AgNO_3_, measuring 30 mL. The mixture was placed in a glass container at room temperature (25 °C) and indoor light conditions while being stirred magnetically. The silver nanoparticles (AgRhNPs) obtained after this process were then centrifuged at 6000 rpm for 1 h. The supernatant was removed, and the nanoparticles were dried in an oven at 45 °C for 24 h. The synthesized silver nanoparticles were weighed and added to ultrapure water with 18.2 MΩ resistivity as a solvent. To get an AgRhNPs stable colloidal dispersion, the nanoparticles in ultrapure water were sonicated for one hour with a final concentration of 1 mg/mL for experiments. In this study, we obtained commercial silver nanoparticles from Sigma-Aldrich (AgCNPs) that were synthesized through chemical means and stabilized using polyvinylpyrrolidone (PVP). We compared their immunomodulatory capacity with those synthesized with *Rumex hym*. extract. The commercial nanoparticles were selected with a morphology and size similar to those produced in our synthesis with *Rumex hymenosepalus*.

### 2.5. UV-Vis Spectroscopy

Spectroscopy was performed in a PerkinElmer Lambda 40 double beam spectrophotometer using the following parameters: Lecture at a range of 900 to 200 nm, at a step speed of 480 nm per min at 25 °C, and a beam aperture width of 0.5 nm. Ultrapure water was used as a blank reference for AgRhNPs and a mixture of ethanol and water (70:30) for Rh extract.

### 2.6. FTIR

Fourier transform IR spectroscopy experiments were performed on a FTIR, System Spectrum GX (PerkinElmer, Shelton, CT, USA). Spectra were obtained in transmittance mode in the region comprised between 4000 and 500 cm^−1^, at a resolution of 0.3 cm^−1^.

### 2.7. TGA

Thermogravimetric analysis was performed on a TGA 7 Thermogravimetric Analyzer (PerkinElmer, Shelton, CT, USA), and samples were carried out at a heating rate of 10° C/min nitrogen atmosphere.

### 2.8. Transmission Electron Microscopy

To analyze the shapes and sizes of AgRhNPs, a 20 µL sample drop was placed on a 300-mesh carbon grid and left to dry at room temperature. The grid was then transferred to a vacuum chamber for 24 h before examination using a JEOL 2010F TEM apparatus (Peabody, MA, USA) with a Gatan CCD camera (Las Positas, CA, USA) attached to the microscope. HRTEM analysis was conducted using Digital Micrograph software (Version 3.7), and size determination of the nanoparticles was performed with ImageJ software (version 1.52a).

### 2.9. Cells Assays

THP-1 cells (ATCC-TIB 202) were cultured in RPMI-1640 complete medium with 2.05 mM L-glutamine, supplemented with 10% heat-inactivated FBS at 37 °C in a 5% CO_2_ atmosphere. For cell assays, THP-1 cells were seeded in 0.5 mL tubes (Eppendorf) at a density of 1 × 10^5^ cells/tube containing RPMI-1640 medium. Once the cell concentration was adjusted briefly for the assay, we added 10 µL of AgRhNPs, Rh extract, AgCNPs, and vehicle (H_2_O miliq). The final concentrations in our AgRhNP system were 6.25 and 12.5 µg/mL, while the concentrations for Rh extract were 25 µg/mL, and for AgCNP, it was 6.875 µg/mL. The vehicle control was H_2_O, and the kill control was THP-1 treated at 70 °C for 45 min. After 6 and 12 h, for the viability assay, we added a dilute calcein-acetoxymethyl (calcein-AM) solution at 5 µM, then incubated for 30 min at 37 °C and read fluorescence at Ex/Em 485/530 nm. For the *Anexxin V-FITC Apoptosis Assay*, THP-1 were cultured in DMEM with 10% FBS until confluence, after which the cells were serum-starved for 6 and 12 h with the same treatments as the viability assay. After treatments, the cells were collected and washed once in 1X phosphate buffered saline (PBS), then once in Annexin V 1X binding buffer (eBioscience, Inc., San Diego, CA, USA), and resuspended in 100 μL of binding buffer 1X (1 × 10^6^/mL), and 1 μL of FITC-conjugated Annexin V (Biolegend, San Diego, CA, USA) was added to the cell suspension and incubated for 10 min in the dark. Cells were washed in 1X binding buffer and resuspended in 200 μL of 1X binding buffer, and 1 × 10^4^ events from region 3 (R3) were analyzed by flow cytometry using a BD FACS Verse flow cytometer (BD Biosciences, Franklin Lakes, NJ, USA). For CD14, CD16, CCR2, Tie-2, and Arg-1 analysis, we used antibodies (ThermoFisher, Waltham, MA, USA) and flow cytometry using a BD FACS Verse flow cytometer (BD Biosciences). Treatments with interleukins were performed, and IL-4, IL-6, and IL-10 markers were used for this assay.

### 2.10. Statistical Analysis

All analysis was performed with the Origin Pro 2018 software, followed by Tukey HSD (Honestly-significant-difference); significant differences are expressed (*p* < 0.05).

## 3. Results and Discussion

### 3.1. Synthesis and Characterization

#### 3.1.1. The Global Profile of Metabolites of Hydroethanolic Rh Extract

**Figure 1:**
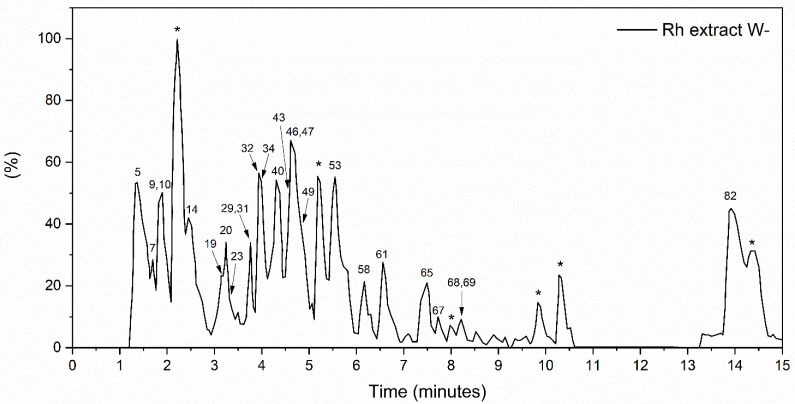
*Rumex hymenosepalus* base peak intensity (BPI) chromatogram in the negative ionization mode. Compounds that could not be identified are marked with *.

Analysis of the global metabolite profile of the *Rumex hymenosepalus* root extract by UPLC-qTOF in negative ionization mode yielded 332 pre-identified compounds, of which 89 met the established identification criteria. These identified compounds are shown in Table S1, ordered according to retention times. Among the compounds, amino acids and peptides, polyphenols (phenolic acids, flavonoids, stilbenes, coumarins, and lignans), organic acids, lipids, and phospholipids, among others, are identified. Flavonoids (flavonols, flavones, isoflavone, chalcones, and anthocyanidin) are the most abundant polyphenols identified in the extract, and several of these compounds are present as Flavonoid Glycosides [30]. Unlike other reports for the genus Rumex, where the presence of anthranoids is abundant [27,31], in our study, only the compound 3,8-Dihydroxy-1-methylanthraquinone-2-carboxylic acid is identified. Table 1 shows the 25 compounds with the highest relative abundance in the extract. The numbers in the first column correspond to the classification of the compound according to the retention time (refer to Table S1). According to the experimental conditions of the UPLC-qTOF assay, compounds with greater polarity correspond to lower numbers, while those with the longest retention time are the least polar. The polyphenolic compounds stand out among the identified compounds, being the flavonoids’ most abundant. Among the flavonoids highlighted by their relative abundance, we have Eriodictyol 7-(6-trans-p-coumaroylglucoside), which is a molecule abundantly found in various medicinal plants, citrus fruits, and vegetables [32] and has a broad spectrum of pharmacological activities. Zhang et al. reported that eriodictyol shows anticancer activity in A549 human lung cancer cells by inducing apoptosis and G2/M cell cycle arrest [33]. Liu and Yan showed that eriodictyol modulates pro-inflammatory cytokines such as tumor necrosis factor (TNF-α), interleukin-6 (IL-6), IL-8, and IL-1β in arthritis fibroblast-like synoviocytes (RA-FLS). Therefore, eriodictyol might be a potential therapeutic agent for the treatment of rheumatoid arthritis [34]. Eriodictyol has a protective effect on various health conditions related to oxidative stress produced by ROS. Oxidative stress-mediated inflammation decreases by either inhibiting PI3K/Akt, FOXO1 signaling, or activating MAPKs, Sirt1 signaling, thus blocking the downstream nuclear translocation of NF-κB [35]. Epiafzelechin 3-O-gallate-(4beta->6)-epigallocatechin 3-O-gallate (EGCG) (Figure 1) is a biflavonoid found in some plants. There are few reports on this compound, and they do not mention any biological activity on human metabolism [36,37,38]. On the contrary, the EGCG therapeutic effects in anticancer treatments are widely known for promoting apoptosis and stopping metastatic processes through the downregulation of nuclear factor κB (NFκB) [39]. EGCG also shows important activity in heart diseases, neurodegenerative diseases such as Parkinson’s and Alzheimer’s, and metabolic disorders such as diabetes mellitus and obesity [40]. The compound (-)-Epicatechin 3-O-gallate (ECG) has marked biological activity due to its anti-inflammatory capacity as an antioxidant agent and anticancer capacity in the stages of angiogenesis and metastasis by regulating cell proliferation and apoptosis. ECG has shown effects on cancer cell lines, mainly in arresting the cell cycle in the G1 phase by modulating the proliferative gene and growth factor and promoting apoptosis by activating the proapoptotic protein [41]. Similarly, studies on HepG2 cell lines showed that ECG could reduce IL-6, which might further modulate acute-phase protein defense against the inflammatory state and enhance host defense against inflammation [42]. It has recently been demonstrated by molecular dynamics studies that the phyto-compound Pavetannin C1 has a high affinity for the spike protein on the surface of the SARS-COV2 virus and can block the binding of this protein to the cell surface [43]. In silico studies by Ksouri et al. indicate that Pavetannin C1 interacts strongly with the amino acid residue Cys145 of the coronavirus protease, favoring this enzyme inhibition and, thus, the replication of the virus [44]. LisoPA phospholipid is enormously essential in various cellular processes. Still, it has also stood out as the phospholipid molecule that increases significantly with diseases such as cancer, Alzheimer’s, etc. It is important to note that efforts are aimed at blocking the receptors for this phospholipid. Resveratrol is one of the molecules proposed as an efficient blocker [45,46]. This molecule is also reported in this extract but not as one of the most abundant; however, we must point out that the concentration is determined based on the interaction with fungi and microorganisms in general since it is a response of the plant’s immune system.

#### 3.1.2. FTIR

**Figure 2:**
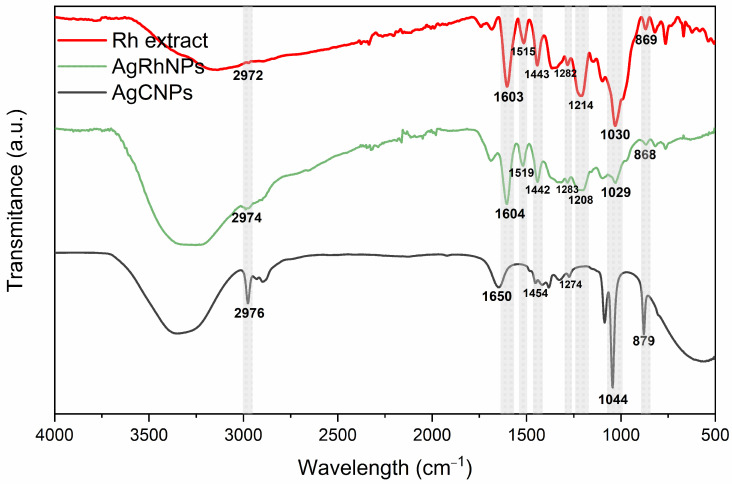
Rh extract, AgRhNPs and AgCNPs FTIR spectra.

FTIR indicated that the *R. hymenosepalus* extract utilized as a reducing agent in the process of nanoparticle synthesis was bound to the surface of AgRhNPs through the formation of phenolic ring bonds. These findings support previous reports of its function as a stabilizer by Rodriguez et al. in 2017 [47]. This analysis, together with TGA, verifies the permanence of post-synthesis compounds; the percentage of organic matter in silver nanoparticles synthesized with *R. hymenosepalus* of 44.5% is also reasonable compared to another synthesis of silver nanoparticles using different extracts of the plant, where they obtained between 30–80% of the organic part [48,49,50]. Figure 2 shows the FTIR spectra of AgCNPs, Rh extract, and AgRhNPs. It showed similar peaks of stretching and deformation from extract molecules. In 2974, 1650, 1603, and 1384, 1351 cm^−1^ correspond respectively to the stretching of bonds of C-H, C=O, and C-O, associated with aromatic rings; also, 1049, 1021, and 799 cm^−1^ are related to the stretching of phenolic rings. The *Rumex hymensosepalus* extract shows a characteristic peak of condensed tannins, which were found in majority form by UHPLC-MS/MS (epicatechin, EGCG, and resveratrol in minor concentrations). By comparison, AgRhNPs preserve the polyphenol peaks and shift the peaks for 1021 cm^−1^ presumably the interaction site between molecules and surface nanoparticles. Figure 2 determines the interaction between biomolecules and nanoparticles, which means that reductor (polyphenolic molecules) maintains the interaction with AgRhNPs. The functionalization of AgRhNPs occurs with modified molecules by the reaction of oxide-reduction oxidized molecules (broadened peak around 1610 cm^−1^), indicating that OH hydroxyl promotes the response [51] and interaction through the C-O group. The AgCNPs stabilized with PVP do not show signals in 1519, 1454, and 1208 cm^−1^ related to functional groups of polyphenols and show a prominent peak in 1044 and 879 cm^−1^ that corresponds with stretching vibration peaks of C-N related to the complexation with PVP [52].

#### 3.1.3. UV-Visible

**Figure 3:**
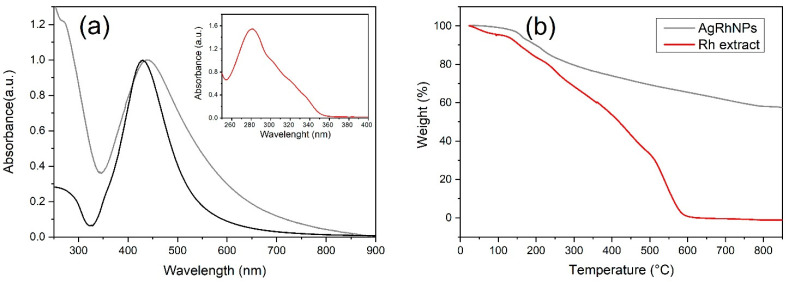
(**a**) UV-Vis of AgRhNPs (gray), AgCNPs (black), and UV-Vis of Rh extract (inset), (**b**) TGA of AgRhNPs (gray) and TGA of Rh extract (red).

The silver nanoparticle UV–Vis absorption spectra are shown in Figure 3a. For AgRhNPs, a well-defined band is observed with a maximum at 435 nm corresponding to the nanoparticles’ localized surface plasmon resonance (LSPR). A small shoulder is also observed in the absorption spectrum at 275 nm associated with the *Rumex hymenosepalus* extract. The inset in Figure 3a corresponds to the absorption spectrum of a Rumex extract aqueous solution with a maximum at 278 nm. This signal is associated with the polyphenolic compounds described in the mass spectroscopy analysis. The above provides further evidence that the final product obtained is a metal-organic material with silver nanoparticles functionalized by extract molecules adsorbed on its surface. It is known that reducing the size of nanoparticles shifts the absorption maximum to higher energies and that the size dispersion is reflected in the half-width of the LSPR [53]. The plasmonic response of AgNPs is also influenced by their morphology. Structures with axial growth exhibit two absorption bands [54,55,56]. In the context of nanoparticle synthesis using plant extracts, some authors have also related this behavior of the absorption bands to an increase in the layers of organic molecules that act as functionalizing agents of the nanoparticles [57]. The present work applied a cleaning process consisting of sonication and centrifugation cycles to the nanoparticle system produced. This cleaning process aims to eliminate the extract from the nanoparticle surface and reduce size polydispersity. The obtained results indicate that the half-width of the resonance plasmon was reduced, but the toxicity of the nanoparticles increased compared to the functionalized nanoparticles. Therefore, for the biological studies, it was chosen to work with the uncleaned nanoparticle system characterized by the absorption spectrum of Figure 3a.

#### 3.1.4. UV-Vis Spectroscopy and Thermogravimetric Analysis

It is necessary to know the metal-organic relationship of our nanoparticle. Thermogravimetric analysis reveals weight variation during the thermic process. Rh extract was vaporized totally at 600 °C (Figure 3b); on the other hand, AgRhNPs lost its organic part at 800 °C, showing us a 55.5% metal and 44.5% organic composition (Figure 3b).

To evaluate the stability of AgRhNP, we compared the UV-Vis spectra and zeta potential of the newly synthesized nanoparticles and the same nanoparticles six months after being stored at room temperature (25 °C). Figure S1 displays the UV-Vis spectra normalized to the resonance plasmon maximum. In both cases, the absorbance maximum is located at 435 nm. However, the absorption at 278 nm, corresponding to the extract, experiences a decrease in intensity from 1.2 to 0.9. The observed variation can be attributed to the removal of extract molecules that were complexed with the particles. This is supported by the data presented in Table S2, which shows changes in the Z-potential of the nanoparticles after being stored at room temperature for six months. The negative surface charge of AgRhNPs is attributed to polyphenolic compounds that are abundant in hydroxyl groups (Figure S2). A reduction in the negative charge indicates the gradual loss of these compounds over time. Despite this, the colloidal dispersion of AgRhNPs remains stable and does not precipitate even after 6 months, as evidenced by its zeta potential value of -26 mV (Figure S3). For therapeutic purposes, it is desirable to have polyphenolic compounds on the surface of the nanoparticle. Therefore, clinical use is recommended immediately after obtaining the synthesis of AgRhNPs [58].

#### 3.1.5. Transmission Electronic Microscopy

**Figure 4:**
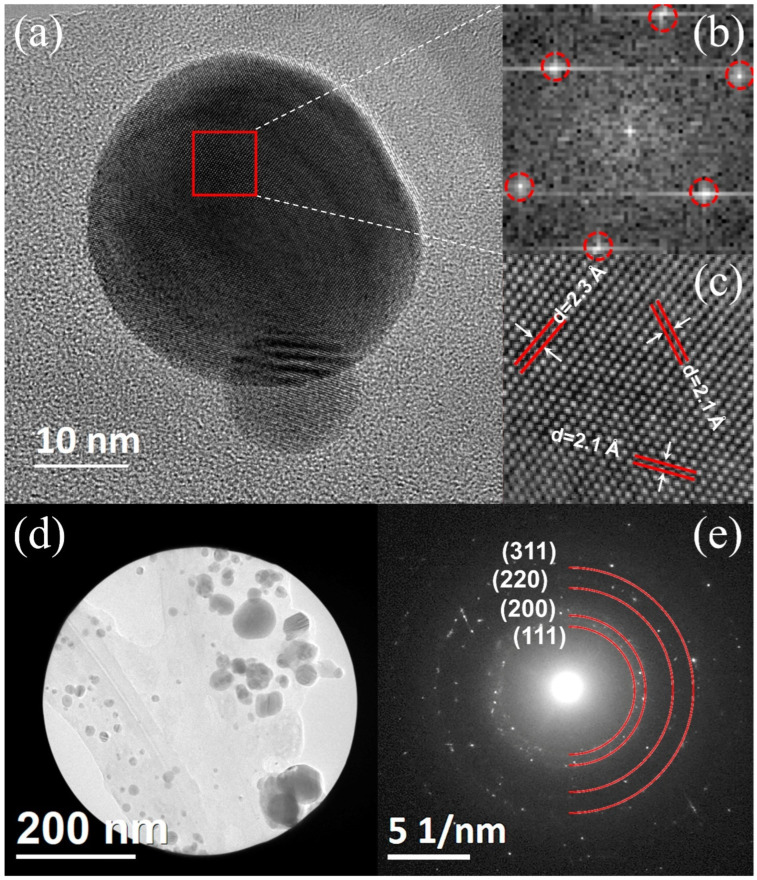
(**a**) AgRhNPs HRTEM micrograph. (**b**) FFT pattern of the selected region. (**c**) The image corresponds to the theoretical reconstruction by inverse FFT of the (**b**) pattern. (**d**) AgRhNPs group and (**e**) their selected area electron diffraction (SAED).

Figure 4a corresponds to the HRTEM of two silver nanoparticles. In Figure 4b, FFT plots from the red square region and their corresponding integrated image from the FFT (Inverse) have interplanar spacing of 2.1 and 2.3 Å (Figure 4c). The crystalline nature of the silver nanoparticles synthesized with *R. hymenosepalus* (Figure 4d) was evaluated by SAED. The corresponding electron diffraction pattern in Figure 4e is associated with the face-centered cubic (fcc) crystal structure of silver [51], with indexes Miller (311), (220), (200), and (111) according to the silver card of the Joint Committee on Powder Diffraction Standards (JCPDS card no. 04-0783) [59].

**Figure 5:**
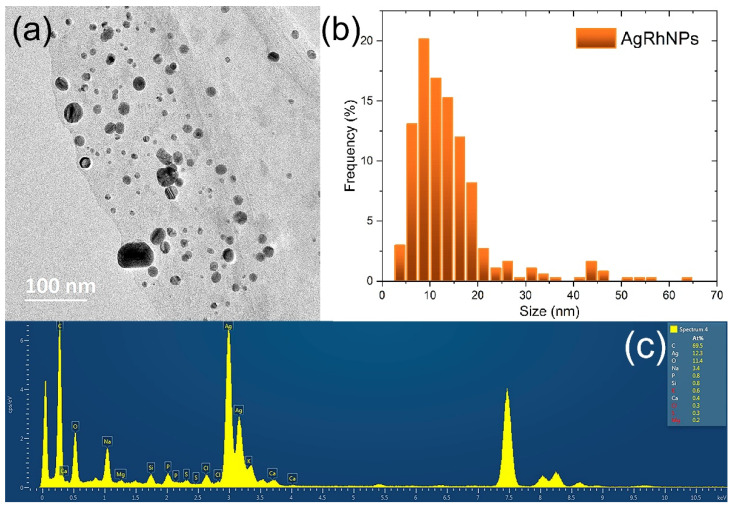
(**a**) Micrograph obtained by TEM. (**b**) Size distribution of AgRhNPs nanoparticles; and (**c**) EDS spectrum corresponding to the nanoparticles shown in (**a**).

Figure 5a TEM analysis confirmed a spherical morphology in AgRhNPs, as well as a polydisperse size with a range of 5 to 26 nm and a mean size of 11 ± 0.3 nm. The size distribution with 500 particles counted (Figure 5b,c) shows characteristics peaking at silver.

### 3.2. Cell Assays

#### 3.2.1. Viability

**Figure 6:**
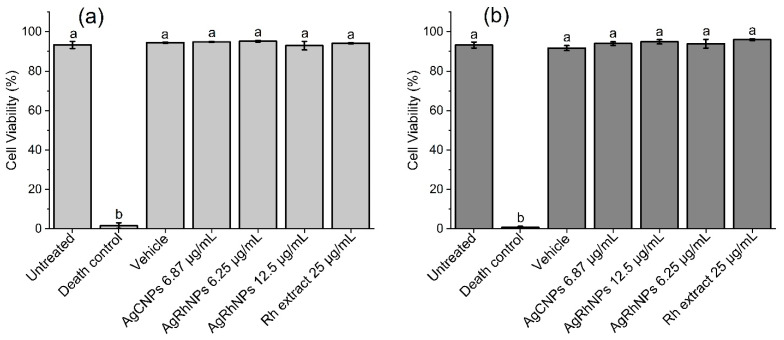
Cell viability assay using calcein-AM. Different treatments were administered to THP-1 and quantified (**a**) at 6 h and (**b**) at 12 h. Both One-way ANOVA and the Tukey test. *p* < 0.05. ^a^ denotes no differences between treatments; ^b^ denotes differences vs. untreated cells.

In Figure 6a, the cell viability assay using calcein-AM for 6 h is about 95% of the viability for all treatments (except Death Control), and similar results were obtained for Figure 6b, the cell viability assay using calcein-AM for 12 h. Corresponding flow cytometry dot plots are shown in Figures S4 and S5. The use of nanomaterials for the benefit of human health from their interaction with monocytes ranges from monocyte inhibition and production to modulation of mobilization, depletion of hyperactive macrophages, modulation of macrophage polarization, biomimetic techniques of nanoparticles, and causing anti-inflammatory effects [60,61,62,63]. By entering nanomaterials in vivo, we will find the immune system, which involves organs, In different works with concentrations of silver nanoparticles similar to ours, a low viability was observed, close to 40%, with ours being greater than 98% viability in all treatments except the death control. The fact that calcein shows that the cell is alive does not indicate whether it is in the process of apoptosis.

#### 3.2.2. Annexin V-FITC Apoptosis Assay

**Figure 7:**
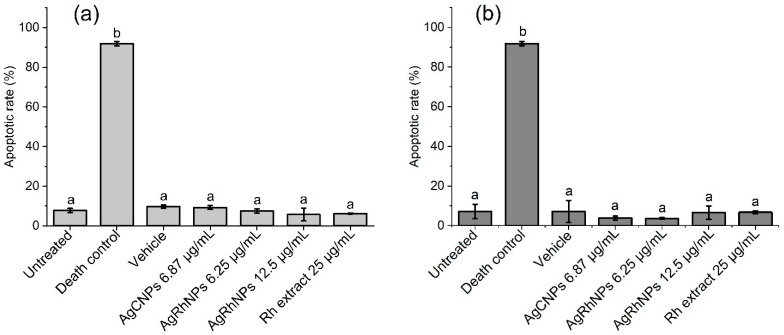
(**a**) Apoptotic rates of THP-1 with treatments for 6 h and (**b**) 12 h determined by an annexin V assay using a flow cytometer. One-way ANOVA with the Tukey test Different superscript letters indicate significant differences between groups (*p* value, *p* ≤ 0.05), ^a^ denotes no differences between treatments, and ^b^ denotes differences from Death Control vs. treatments.

In Figure 7a,b, all treatments except Death Control gave an apoptotic rate of less than 10% with no significant difference at 6 and 12 h, respectively. Various authors state that silver nanoparticles affect the integrity of the membrane in THP-1 [64], causing a series of molecular events and leading to cell death, so we performed the Annexin V-FITC apoptosis assay, where it can be seen that none of the treatments caused an apoptotic event with a significant difference from the control at 6 and 12 h of interaction, except for the death control.

#### 3.2.3. Monocytes Subsets

**Figure 8:**
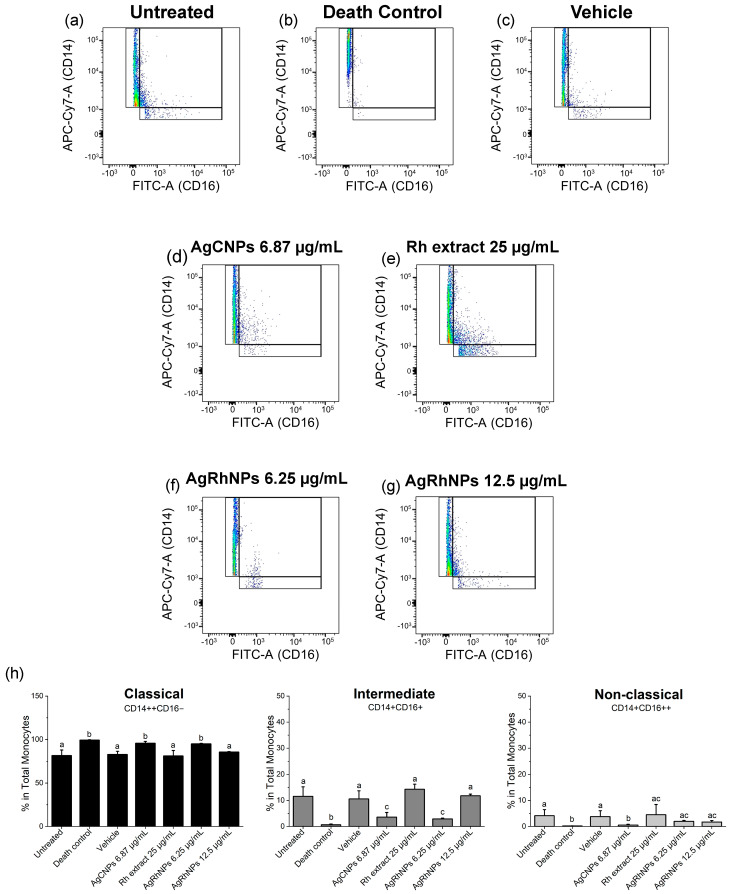
Polarization of monocyte subsets after interaction with treatments (**a**–**g**) Dots of treatments (**h**) Representative density plots for classical, intermediate, and non-classical monocyte polarization after 12 h of challenge One-way ANOVA with the Tukey test Different superscript letters indicate significant differences between groups (*p* value, *p* ≤ 0.05).

Due to the fact that the membrane glycoprotein CD14 is greatly expressed on the surface of human monocytes, it serves as a label when working with human monocytes, in turn being able to catalog monocytes depending on protein expression, such as CD14++CD16− classic, CD14+CD16+ intermediate, and CD14+CD16++ non-classic [65]. The contrast between the treatments when expressing the monocyte subset can be noted in Figure 8. On the one hand, the untreated vehicle, *Rumex*, and concentration of 12.5 µg/mL of AgRhNPs present a normal expression of classic monocytes that can differentiate into macrophages in tissue [66], sharing the expression of intermediate monocytes, which are associated with an interface between classic and non-classical [67]. The discussion continues regarding its anti-inflammatory and phagocytic properties. In treatments, the expression trend is the same in non-classical monocytes, as various authors speak of its anti-inflammatory capacity [68].

**Figure 9:**
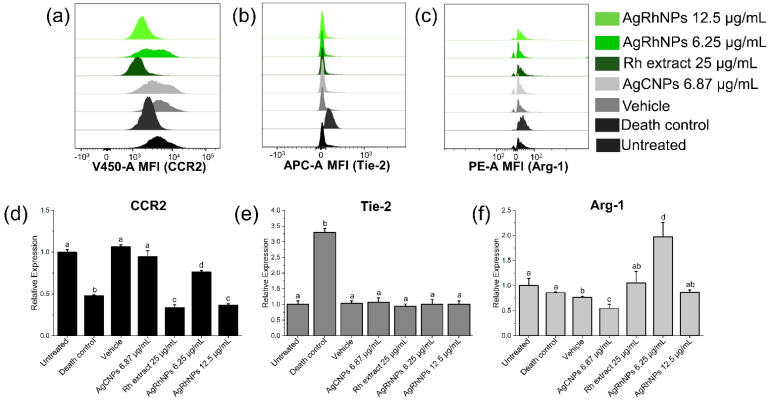
Polarization of monocyte subsets after extracting stimuli with treatments (**a**) Representative density plots for CCR2, (**b**) Tie-2, and (**c**) Arg-1 expression after 12 hours of challenge with treatments. (**d**) Representative histograms of CCR2, (**e**) Tie-2, and (**f**) Arg-1 expression in monocytes measured as mean fluorescence intensity units (MFI) after 12 h of challenge with treatments. One-way ANOVA with the Tukey test Different superscript letters indicate significant differences between groups (*p* value, *p* ≤ 0.05).

According to Figure 9, monocytes treated with AgRhNPs (12.5 µg/mL) and Rh extract (25 µg/mL) exhibit significantly reduced levels of the CC chemokine receptor type 2 (CCR2), which is accompanied by the classical monocytes CD14++ CD16−, compared to the other treatments. CCR2, having high expression, has been involved in pathologies such as atherosclerosis and myocardial infarction [69,70,71] and has become the target of therapy to prevent systemic inflammation [72]. Tie-2, despite being currently a target of study, is expressed in monocytes and, when inhibited, thereby causes a decrease in angiogenesis, which prevents tumor growth [73,74,75]. There is no information on studies of nanoparticles with this receptor, and in our investigation, no significant difference was observed between the treatments. Arg-1 is involved in hydrolyzing L-arginine into urea and L-ornithine [76,77]. It was observed that the concentration of 6.25 µg/mL of AgRhNPs obtained a significantly high expression, unlike the other treatments. Arg-1 deficiency affects the urea cycle of the liver, causing, among other things, neurological damage accompanied by progressive intellectual loss as well as regulating nitric oxide in the body [78,79].

#### 3.2.4. Interleukins Production of THP1 Cells

Silver nanoparticles represent an innovative proposal in the biomedical field. However, their proven cytotoxicity can be a difficulty if we use these particles as drug carriers or diagnostic tools due to the immunocompatibility and immunotoxicity evidenced by various authors [80,81]. Interleukins are responsible for controlling the differentiation and proliferation of some cell subpopulations [61], activating the endothelium and increasing vascular permeability, facilitating the migration of immune cells from the bloodstream to the tissue, promoting the secretion of antibodies, and controlling the T lymphocyte response [61,82].

Parnsamut & Brimson, 2015, evidenced the inhibitory effect of AgNPs on the production of pro-inflammatory cytokines IL-2, IL6, and TNF-α in human leukemia cell lines, both in pro-monocytes U937 and T lymphocytes Jurkat [83]; on the other hand, Murphy et al. (2016), using cultures of THP-1 monocytes and human monocytes isolated from the peripheral blood of healthy donors, observed that AgNPs induce an innate immune response through the up-regulation of these inflammatory cytokines IL-1, IL-6, and TNF-α [84].

**Figure 10:**
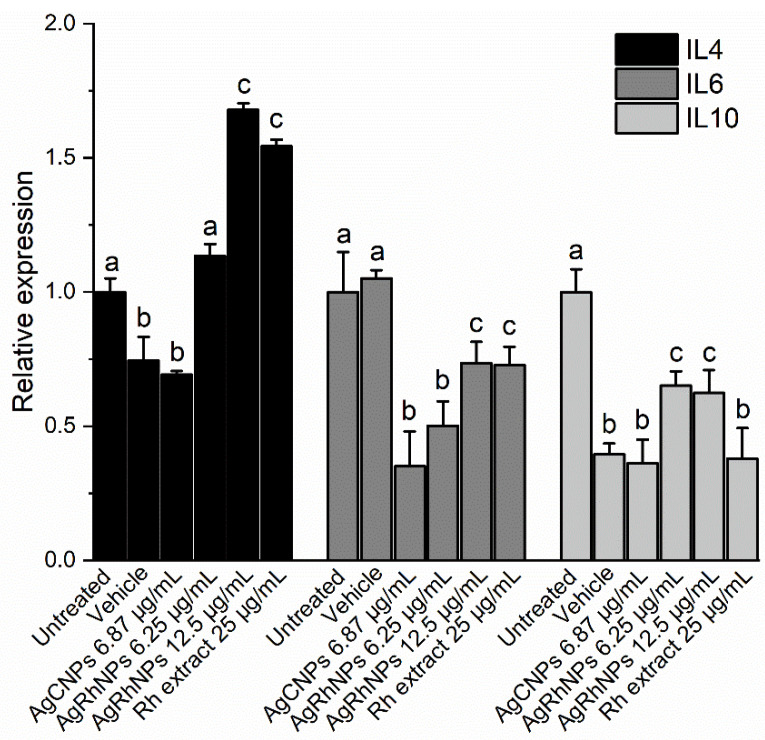
Determination of Cytokines levels and relative expression of IL4, IL6, and IL10 after 12 h of challenge with treatment One-way ANOVA with the Tukey test Different superscript letters indicate significant differences between groups (*p* value, *p* ≤ 0.05).

In the present study, we observed that both AgCNPs and AgRhNPs significantly inhibit the production of IL-6 in THP-1 cells at concentrations of 6.25 and 12.5 µg/mL (Figure 10). A similar effect is also observed in THP-1 stimulated with nanoparticles encapsulated in liposomes by Yusuf & Casey (2019), who demonstrated that the encapsulation of AgNPs suppresses the inflammatory response of THP-1 [81]. With encapsulation, it is possible to use these without adverse effects. This suppressive effect of IL-6 given by the encapsulation of the NPs could be comparable to that shown here when carrying out a green synthesis process. The final product obtained is a metallic particle coated with bioactive molecules from Rh extract. Thus, in the first instance, the cell interacts with these compounds instead of the metallic part, reducing its pro-inflammatory effect. Similarly, flow cytometry showed that both AgRhNPs and Rumex extract independently induce higher production of the anti-inflammatory cytokine IL-4 compared to basal state cells. In contrast, AgCNPs exhibit lower values than the cell control without stimulation (Figure 10). Notably, treatment with AgRhNPs at 12.5 µg/mL and Rh extract at 25 µg/mL elicits the same IL-4 production. According to TGA analysis, AgRhNPs possess a metallic content of 55% (6.875 µg/mL) and an organic content of 45% (5.625 µg/mL). This indicates that the identical outcome is attainable with only one-fifth of the Rh extract treatment content (25 µg/mL). This synergy between the metallic and organic components in the AgRhNPs system may be associated with their efficient internalization into cells compared to a freely administered extract. Other investigations with the THP-1 monocytic cell line showed no induction of IL-4 production by AgNPs obtained through the reduction of silver nitrate with sodium borohydride [85]. Furthermore, it was observed that IL-4 production decreased in the lungs of mice that were chronically exposed to AgNPs and had metabolic syndrome [86].

Galbiati et al. (2018) observed that in THP-1 monocytes stimulated with AgNPs, there is no variation in the expression of IL-10 with or without treatment with Lipopolysaccharide (LPS) [80]. In our study, there was a decrease in the production of the anti-inflammatory cytokine IL-10 in monocytes stimulated with AgRhNPs compared to those not stimulated (Figure 10). IL-10 and IL-4 are essential anti-inflammatory cytokines with the ability to regulate macrophages and dendritic cells and also act as responsible for the proliferation and differentiation of Th2 cells [61,87]; However, by stimulating THP-1 monocytes with AgRhNPs obtained by green synthesis, we can observe that they could be modulating an anti-inflammatory effect by decreasing the production of IL-6 and increasing the production of IL-4 exclusively, evidencing that the presence of these NPs confers anti-inflammatory properties.

Some of the principal compounds from *Rumex hymenosepalus* (Table 1), like gallic acid, carthamin, procyanidin 1, and galangin, have reports about their effects on decreasing the expression of IL-6 [88,89,90,91]. It is also reported that Procyanidin 1, eriodictyol, and galangin induce the release of cytokines that reduce inflammation, such as IL-4 and IL-10 [91,92,93].

## 4. Conclusions

The UPLC-qTOF analysis of the global metabolite profile of Rumex hymenosepalus root extract reveals a substantial presence of antioxidant compounds, particularly flavonoids. These compounds enable the efficient synthesis of silver nanoparticles by functioning as reducing agents of Ag+ ions and subsequently acting as stabilizing agents adsorbed on the surface of the formed particles. The AgRhNPs nanoparticles and Rh extract neither exhibited cytotoxicity in the THP-1 monocyte cell line. Additionally, the treatments mentioned above exhibited anti-inflammatory effects by maintaining the classical monocyte phenotype CD14++CD16, reducing pro-inflammatory interleukin IL-6 production, and increasing IL-4 production. Conversely, silver nanoparticles produced with citrate and stabilized with PVP did not exhibit these immunomodulatory effects, implying that the extract’s compounds are necessary for this response. The TGA analysis results and IL production suggest that there could be synergistic effects between the particle’s metallic content and its organic content on the surface. Based on all the evidence, AgRhNPs and Rh extract may be beneficial in chronic inflammatory processes due to their biocompatibility and immunostimulatory capabilities, so it is necessary to carry out in vivo studies to support this proposal.

## Figures and Tables

**Table 1:** Most abundant compounds in negative ionization mode.

No.	Accepted Description	Neutral Mass (Da)	*m*/*z*	Adducts	Retention Time (min)	Formula
34	Eriodictyol 7-(6-trans-p-coumaroylglucoside)	596.15	577.13	M-H_2_O-H	3.96	C_30_H_28_O_13_
61	Epiafzelechin 3-O-gallate-(4beta->6)-epigallocatechin 3-O-gallate	882.16	881.15	M-H	6.56	C_44_H_34_O_20_
53	(-)-Epicatechin 3-O-gallate	442.09	441.08	M-H	5.56	C_22_H_18_O_10_
23	Pavetannin C1	1152.25	1151.23	M-H	3.34	C_60_H_48_O_24_
88	LysoPA(0:0/18:2(9Z,12Z))	434.24	433.24	M-H	18.06	C_21_H_39_O_7_P
58	6-{4-[(1E)-3-{3-[6-carboxy-5-(2,4-dihydroxyphenyl)-3-methylcyclohex-2-en-1-yl]-2,4-dihydroxyphenyl}-3-oxoprop-1-en-1-yl]-3-hydroxyphenoxy}-3,4,5-trihydroxyoxane-2-carboxylic acid	694.18	729.15	M+Cl	6.18	C_35_H_34_O_15_
82	Galangin	270.05	269.05	M-H	13.91	C_15_H_10_O_5_
47	Rutarin	424.14	405.12	M-H_2_O-H	4.63	C_20_H_24_O_10_
46	9,10-dihydroxy-8,8-dimethyl-2H,8H,9H,10H-pyrano[2,3-h]chromen-2-one	262.08	243.07	M-H_2_O-H	4.63	C_14_H_14_O_5_
14	3-(4-hydroxy-3-methoxyphenyl)oxirane-2-carboxylic acid	210.05	255.05	M+FA-H	2.47	C_10_H_10_O_5_
20	6-[4-(2-carboxyeth-1-en-1-yl)-5-hydroxy-2-methoxyphenoxy]-3,4,5-trihydroxyoxane-2-carboxylic acid	386.09	407.07	M+Na-2H	3.26	C_16_H_18_O_11_
40	Marshrin	290.08	289.07	M-H	4.40	C_15_H_14_O_6_
32	Procyanidin C1	866.21	865.20	M-H	3.88	C_45_H_38_O_18_
19	2-{[5,7-dihydroxy-2-(4-hydroxyphenyl)-4-oxo-4H-chromen-3-yl]oxy}-4,5-dihydroxy-6-methyloxan-3-yl (2E)-3-(4-hydroxyphenyl)prop-2-enoate	578.14	577.14	M-H	3.21	C_30_H_26_O_12_
7	2-Galloylglucose	332.07	331.07	M-H	1.67	C_13_H_16_O_10_
49	Aegelinol	246.09	227.07	M-H_2_O-H	4.81	C_14_H_14_O_4_
29	6-[(E)-2-(2H-1,3-benzodioxol-5-yl)ethenyl]-5-hydroxy-4-methoxy-5,6-dihydro-2H-pyran-2-one	290.08	289.07	M-H	3.78	C_15_H_14_O_6_
43	Epicatechin 3-O-gallate-(4beta->6)-epicatechin 3-O-gallate-(4beta->8)-catechin	1170.23	1169.21	M-H	4.50	C_59_H_46_O_26_
5	2,5-Dioxopentanoate	130.03	111.01	M-H_2_O-H	1.41	C_5_H_6_O_4_
31	Carthamin	910.22	891.20	M-H_2_O-H	3.78	C_43_H_42_O_22_
10	Gallic acid	170.02	169.02	M-H	1.98	C_7_H_6_O_5_
65	5,6-Dihydro-5-hydroxy-6-methyl-2H-pyran-2-one (5,6-dehydrokawain)	228.08	227.07	M-H	7.49	C_14_H_12_O_3_
9	3-Methylglutaconic acid	144.04	125.03	M-H_2_O-H	1.98	C_6_H_8_O_4_
69	Quercetin	302.05	301.04	M-H	8.21	C_15_H_10_O_7_
68	5-(3-methoxyphenyl)-4-(sulfooxy)pentanoic acid	304.06	285.04	M-H_2_O-H	8.21	C_12_H_16_O_7_S
67	Aloesol 7-glucoside	378.14	377.13	M-H	7.72	C_19_H_24_O_9_

## Data Availability

Data are contained within the article.

## Supplementary Materials

The supporting information can be downloaded at: https://www.mdpi.com/article/10.3390/nano14010106/s1. References [94,95,96,97] are cited in the supplementary materials.

## References

[1] Mulvaney P. (2015). Nanoscience vs Nanotechnology—Defining the Field. ACS Nano.

[2] Hulla J.E., Sahu S.C., Hayes A.W. (2015). Nanotechnology: History and Future. Hum. Exp. Toxicol..

[3] Kumar A., Shah S.R., Jayeoye T.J., Kumar A., Parihar A., Prajapati B., Singh S., Kapoor D.U. (2023). Biogenic Metallic Nanoparticles: Biomedical, Analytical, Food Preservation, and Applications in Other Consumable Products. Front. Nanotechnol..

[4] Khan A., Roy A., Bhasin S., Emran T.B., Khusro A., Eftekhari A., Moradi O., Rokni H., Karimi F. (2022). Nanomaterials: An Alternative Source for Biodegradation of Toxic Dyes. Food Chem. Toxicol..

[5] Na J., Zheng D., Kim J., Gao M., Azhar A., Lin J., Yamauchi Y. (2022). Material Nanoarchitectonics of Functional Polymers and Inorganic Nanomaterials for Smart Supercapacitors. Small.

[6] Babu P.J., Tingirikari J.M.R. (2023). A Review on Polymeric Nanomaterials Intervention in Food Industry. Polym. Bull..

[7] Yang X., Yang M., Pang B., Vara M., Xia Y. (2015). Gold Nanomaterials at Work in Biomedicine. Chem. Rev..

[8] Nguyen N.H.A., Falagan-Lotsch P. (2023). Mechanistic Insights into the Biological Effects of Engineered Nanomaterials: A Focus on Gold Nanoparticles. Int. J. Mol. Sci..

[9] Metal Nanoparticles Global Market Trends, Growth Analysis, Outlook 2032. https://www.thebusinessresearchcompany.com/report/metal-nanoparticles-global-market-report.

[10] Arroyo G.V., Madrid A.T., Gavilanes A.F., Naranjo B., Debut A., Arias M.T., Angulo Y. (2020). Green Synthesis of Silver Nanoparticles for Application in Cosmetics. J. Environ. Sci. Health A Tox. Hazard. Subst. Environ. Eng..

[11] Vinod T.P., Jelinek R. (2019). Inorganic Nanoparticles in Cosmetics. Nanocosmetics.

[12] Szczepańska E., Bielicka-Giełdoń A., Niska K., Strankowska J., Żebrowska J., Inkielewicz-Stępniak I., Łubkowska B., Swebocki T., Skowron P., Grobelna B. (2020). Synthesis of Silver Nanoparticles in Context of Their Cytotoxicity, Antibacterial Activities, Skin Penetration and Application in Skincare Products. Supramol. Chem..

[13] Shamaila S., Jalil A., Ishfaq M., Sharif R. (2022). Nano-Technological Aspects of Zinc Oxide and Silver in Cosmetics. J. Appl. Phys..

[14] Ediyilyam S., George B., Shankar S.S., Dennis T.T., Wacławek S., Černík M., Padil V.V.T. (2021). Chitosan/Gelatin/Silver Nanoparticles Composites Films for Biodegradable Food Packaging Applications. Polymers.

[15] de Oliveira Morais L., Macedo E.V., Granjeiro J.M., Delgado I.F. (2020). Critical Evaluation of Migration Studies of Silver Nanoparticles Present in Food Packaging: A Systematic Review. Crit. Rev. Food Sci. Nutr..

[16] Moradi M., Razavi R., Omer A.K., Farhangfar A., McClements D.J. (2022). Interactions between Nanoparticle-Based Food Additives and Other Food Ingredients: A Review of Current Knowledge. Trends Food Sci. Technol..

[17] Shi C., Pamer E.G. (2011). Monocyte Recruitment during Infection and Inflammation. Nat. Rev. Immunol..

[18] Libby P. (2006). Inflammation and Cardiovascular Disease Mechanisms. Am. J. Clin. Nutr..

[19] Lemus-de la Cruz J., Trejo-Hurtado M., Landa-Moreno C., Peña-Montes D., Landeros-Páramo J.L., Cortés-Rojo C., Montoya-Pérez R., Rosas G., Saavedra-Molina A. (2023). Antioxidant Effects of Silver Nanoparticles Obtained by Green Synthesis from the Aqueous Extract of Eryngium Carlinae on the Brain Mitochondria of Streptozotocin-Induced Diabetic Rats. J. Bioenerg. Biomembr..

[20] Alkhalaf M.I., Hussein R.H., Hamza A. (2020). Green Synthesis of Silver Nanoparticles by Nigella Sativa Extract Alleviates Diabetic Neuropathy through Anti-Inflammatory and Antioxidant Effects. Saudi J. Biol. Sci..

[21] Jini D., Sharmila S. (2020). Green Synthesis of Silver Nanoparticles from Allium Cepa and Its in Vitro Antidiabetic Activity. Mater. Today.

[22] Khalaf Y.H., Dawood Y., Khashan A.A. (2023). Green Biosynthesis of Berberine-Mediated Silver Nanorods: Their Protective and Antidiabetic Effects in Streptozotocin-Induced Diabetic Rats. Results Chem..

[23] Karuppannan P., Saravanan K., Ashokkumar M., Egbuna C. (2023). Facile Green Synthesis of Silver Nanoparticles Using Ventilago Maderaspatana Leaf Extract, Physicochemical Properties and Evaluation of Antidiabetic Potential against Streptozotocin Induced Diabetic Albino Rats. Res. Sq..

[24] Ullah S., Shah S.W.A., Qureshi M.T., Hussain Z., Ullah I., Kalsoom U.-E., Rahim F., Rahman S.S.U., Sultana N., Khan M.K. (2021). Antidiabetic and Hypolipidemic Potential of Green AgNPs against Diabetic Mice. ACS Appl. Bio Mater..

[25] Vijayaraghavan K., Ashokkumar T. (2017). Plant-Mediated Biosynthesis of Metallic Nanoparticles: A Review of Literature, Factors Affecting Synthesis, Characterization Techniques and Applications. J. Environ. Chem. Eng..

[26] Kharissova O.V., Dias H.V.R., Kharisov B.I., Pérez B.O., Pérez V.M.J. (2013). The Greener Synthesis of Nanoparticles. Trends Biotechnol..

[27] Li J.-J., Li Y.-X., Li N., Zhu H.-T., Wang D., Zhang Y.-J. (2022). The Genus Rumex (Polygonaceae): An Ethnobotanical, Phytochemical and Pharmacological Review. Nat. Prod. Bioprospect..

[28] VanderJagt T.J., Ghattas R., VanderJagt D.J., Crossey M., Glew R.H. (2002). Comparison of the Total Antioxidant Content of 30 Widely Used Medicinal Plants of New Mexico. Life Sci..

[29] Kanazawa M., Ninomiya I., Hatakeyama M., Takahashi T., Shimohata T. (2017). Microglia and Monocytes/Macrophages Polarization Reveal Novel Therapeutic Mechanism against Stroke. Int. J. Mol. Sci..

[30] Bartnik M., Facey P.C., Badal S., Delgoda R. (2017). Glycosides. Pharmacognosy.

[31] Li Y.-X., Li N., Li J.-J., Zhang M., Zhu H.-T., Wang D., Zhang Y.-J. (2022). New Seco-Anthraquinone Glucoside from the Roots of Rumex Crispus. Nat. Prod. Bioprospect..

[32] He P., Yan S., Wen X., Zhang S., Liu Z., Liu X., Xiao C. (2019). Eriodictyol Alleviates Lipopolysaccharide-Triggered Oxidative Stress and Synaptic Dysfunctions in BV-2 Microglial Cells and Mouse Brain. J. Cell. Biochem..

[33] Zhang Y., Zhang R., Ni H. (2020). Eriodictyol Exerts Potent Anticancer Activity against A549 Human Lung Cancer Cell Line by Inducing Mitochondrial-Mediated Apoptosis, G2/M Cell Cycle Arrest and Inhibition of m-TOR/PI3K/Akt Signalling Pathway. Arch. Med. Sci..

[34] Liu Y., Yan X. (2019). Eriodictyol Inhibits Survival and Inflammatory Responses and Promotes Apoptosis in Rheumatoid Arthritis Fibroblast-like Synoviocytes through AKT/FOXO1 Signaling. J. Cell. Biochem..

[35] Islam A., Islam M.S., Rahman M.K., Uddin M.N., Akanda M.R. (2020). The Pharmacological and Biological Roles of Eriodictyol. Arch. Pharm. Res..

[36] Li M., Shen Y., Ling T., Ho C.-T., Li D., Guo H., Xie Z. (2021). Analysis of Differentiated Chemical Components between Zijuan Purple Tea and Yunkang Green Tea by UHPLC-Orbitrap-MS/MS Combined with Chemometrics. Foods.

[37] Xiong F., Nie X., Yang L., Wang L., Li J., Zhou G. (2021). Non-Target Metabolomics Revealed the Differences between *Rh. Tanguticum* Plants Growing under Canopy and Open Habitats. BMC Plant Biol..

[38] Wong C., Ling Y.S., Wee J.L.S., Mujahid A., Müller M. (2020). A Comparative UHPLC-Q/TOF-MS-Based Eco-Metabolomics Approach Reveals Temperature Adaptation of Four Nepenthes Species. Sci. Rep..

[39] Saeki K., Hayakawa S., Nakano S., Ito S., Oishi Y., Suzuki Y., Isemura M. (2018). In Vitro and in Silico Studies of the Molecular Interactions of Epigallocatechin-3-O-Gallate (EGCG) with Proteins That Explain the Health Benefits of Green Tea. Molecules.

[40] Mokra D., Joskova M., Mokry J. (2022). Therapeutic Effects of Green Tea Polyphenol (-)-Epigallocatechin-3-Gallate (EGCG) in Relation to Molecular Pathways Controlling Inflammation, Oxidative Stress, and Apoptosis. Int. J. Mol. Sci..

[41] Li Z., Feng C., Dong H., Jin W., Zhang W., Zhan J., Wang S. (2022). Health Promoting Activities and Corresponding Mechanism of (–)-Epicatechin-3-Gallate. Food Sci. Hum. Wellness.

[42] el-Saadany M.A., Rawel H.M., Raila J., el-Dashloty M.S., Schweigert F.J. (2008). Antioxidants Modulate the IL-6 Induced Inhibition of Negative Acute-Phase Protein Secretion in HepG2 Cells. Cell Biochem. Funct..

[43] Prasanth D.S.N.B.K., Murahari M., Chandramohan V., Panda S.P., Atmakuri L.R., Guntupalli C. (2021). In Silico Identification of Potential Inhibitors from Cinnamon against Main Protease and Spike Glycoprotein of SARS CoV-2. J. Biomol. Struct. Dyn..

[44] Ksouri A., Klouz A., Bouhaouala-Zahar B., Moussa F., Bezzarga M. (2023). Docking-Based Evidence for the Potential of ImmunoDefender: A Novel Formulated Essential Oil Blend Incorporating Synergistic Antiviral Bioactive Compounds as Promising Mpro Inhibitors against SARS-CoV-2. Molecules.

[45] Ferraresi A., Esposito A., Girone C., Vallino L., Salwa A., Ghezzi I., Thongchot S., Vidoni C., Dhanasekaran D.N., Isidoro C. (2021). Resveratrol Contrasts LPA-Induced Ovarian Cancer Cell Migration and Platinum Resistance by Rescuing Hedgehog-Mediated Autophagy. Cells.

[46] Guo K., Feng Y., Zheng X., Sun L., Wasan H.S., Ruan S., Shen M. (2021). Resveratrol and Its Analogs: Potent Agents to Reverse Epithelial-to-Mesenchymal Transition in Tumors. Front. Oncol..

[47] Mollahosseini A., Rahimpour A., Jahamshahi M., Peyravi M., Khavarpour M. (2012). The Effect of Silver Nanoparticle Size on Performance and Antibacteriality of Polysulfone Ultrafiltration Membrane. Desalination.

[48] Wei L., Lu J., Xu H., Patel A., Chen Z.S., Chen G. (2015). Silver Nanoparticles: Synthesis, Properties, and Therapeutic Applications. Drug Discov. Today.

[49] Sharma V.K., Yngard R.A., Lin Y. (2009). Silver Nanoparticles: Green Synthesis and Their Antimicrobial Activities. Adv. Colloid Interface Sci..

[50] Baliah N.T., Muthulakshmi P., Sheeba P.C., Priyatharsini S.L. (2018). Green Synthesis and Characterization of Nanocomposites. Int. Res. J. Eng. Technol..

[51] Das D., Ghosh R., Mandal P. (2019). Biogenic Synthesis of Silver Nanoparticles Using S1 Genotype of Morus Alba Leaf Extract: Characterization, Antimicrobial and Antioxidant Potential Assessment. SN Appl. Sci..

[52] Zhang Z., Zhang X., Xin Z., Deng M., Wen Y., Song Y. (2011). Synthesis of Monodisperse Silver Nanoparticles for Ink-Jet Printed Flexible Electronics. Nanotechnology.

[53] Kang H., Buchman J.T., Rodriguez R.S., Ring H.L., He J., Bantz K.C., Haynes C.L. (2019). Stabilization of Silver and Gold Nanoparticles: Preservation and Improvement of Plasmonic Functionalities. Chem. Rev..

[54] Marinescu L., Ficai D., Ficai A., Oprea O., Nicoara A.I., Vasile B.S., Boanta L., Marin A., Andronescu E., Holban A.-M. (2022). Comparative Antimicrobial Activity of Silver Nanoparticles Obtained by Wet Chemical Reduction and Solvothermal Methods. Int. J. Mol. Sci..

[55] Marinescu L., Ficai D., Oprea O., Marin A., Ficai A., Andronescu E., Holban A.-M. (2020). Optimized Synthesis Approaches of Metal Nanoparticles with Antimicrobial Applications. J. Nanomater..

[56] Gherasim O., Puiu R.A., Bîrcă A.C., Burdușel A.-C., Grumezescu A.M. (2020). An Updated Review on Silver Nanoparticles in Biomedicine. Nanomaterials.

[57] Khan I., Saeed K., Khan I. (2019). Nanoparticles: Properties, Applications and Toxicities. Arab. J. Chem..

[58] Abdel-Aty A.M., Barakat A.Z., Bassuiny R.I., Mohamed S.A. (2023). Statistical Optimization, Characterization, Antioxidant and Antibacterial Properties of Silver Nanoparticle Biosynthesized by Saw Palmetto Seed Phenolic Extract. Sci. Rep..

[59] Vazquez-Muñoz R., Arellano-Jimenez M.J., Lopez F.D., Lopez-Ribot J.L. (2019). Protocol Optimization for a Fast, Simple and Economical Chemical Reduction Synthesis of Antimicrobial Silver Nanoparticles in Non-Specialized Facilities. BMC Res. Notes.

[60] Murray P.J. (2017). Macrophage Polarization. Annu. Rev. Physiol..

[61] Shapouri-Moghaddam A., Mohammadian S., Vazini H., Taghadosi M., Esmaeili S.A., Mardani F., Seifi B., Mohammadi A., Afshari J.T., Sahebkar A. (2018). Macrophage Plasticity, Polarization, and Function in Health and Disease. J. Cell. Physiol..

[62] Dinarello C.A. (2010). Anti-Inflammatory Agents: Present and Future. Cell.

[63] Gaspar N., Zambito G., Löwik C.M.W.G., Mezzanotte L. (2019). Active Nano-Targeting of Macrophages. Curr. Pharm. Des..

[64] Poupot R., Goursat C., Fruchon S. (2018). Multivalent Nanosystems: Targeting Monocytes/Macrophages. Int. J. Nanomed..

[65] Ziegler-Heitbrock L., Ancuta P., Crowe S., Dalod M., Grau V., Hart D.N., Leenen P.J.M., Liu Y.-J., MacPherson G., Randolph G.J. (2010). Nomenclature of Monocytes and Dendritic Cells in Blood. Blood.

[66] Yang J., Zhang L., Yu C., Yang X.-F., Wang H. (2014). Monocyte and Macrophage Differentiation: Circulation Inflammatory Monocyte as Biomarker for Inflammatory Diseases. Biomark. Res..

[67] Merah-Mourah F., Cohen S.O., Charron D., Mooney N., Haziot A. (2020). Identification of Novel Human Monocyte Subsets and Evidence for Phenotypic Groups Defined by Interindividual Variations of Expression of Adhesion Molecules. Sci. Rep..

[68] Narasimhan P.B., Marcovecchio P., Hamers A.A.J., Hedrick C.C. (2019). Nonclassical Monocytes in Health and Disease. Annu. Rev. Immunol..

[69] França C.N., Izar M.C., Hortêncio M.N., Amaral J.B., Ferreira C.E., Tuleta I.D., Fonseca F.A. (2017). Monocyte Sub-Types and the CCR2 Chemokine Receptor in Cardiovascular Disease. Clin. Sci..

[70] Serbina N.V., Pamer E.G. (2006). Monocyte Emigration from Bone Marrow during Bacterial Infection Requires Signals Mediated by Chemokine Receptor CCR2. Nat. Immunol..

[71] Xia M., Sui Z. (2009). Recent Developments in CCR2 Antagonists. Expert Opin. Ther. Pat..

[72] Jabir M.S., Saleh Y.M., Sulaiman G.M., Yaseen N.Y., Sahib U.I., Dewir Y.H., Alwahibi M.S., Soliman D.A. (2021). Green Synthesis of Silver Nanoparticles Using Annona Muricata Extract as an Inducer of Apoptosis in Cancer Cells and Inhibitor for NLRP3 Inflam-Masome via Enhanced Autophagy. Nanomaterials.

[73] De Palma M., Murdoch C., Venneri M.A., Naldini L., Lewis C.E. (2007). Tie2-Expressing Monocytes: Regulation of Tumor Angiogenesis and Therapeutic Implications. Trends Immunol..

[74] Turrini R., Pabois A., Xenarios I., Coukos G., Delaloye J.-F., Doucey M.-A. (2017). TIE-2 Expressing Monocytes in Human Cancers. Oncoimmunology.

[75] Bron S., Henry L., Faes-Van’t Hull E., Turrini R., Vanhecke D., Guex N., Ifticene-Treboux A., Marina Iancu E., Semilietof A., Rufer N. (2016). TIE-2-Expressing Monocytes Are Lymphangiogenic and Associate Specifically with Lymphatics of Human Breast Cancer. Oncoimmunology.

[76] Durante W., Johnson F.K., Johnson R.A. (2007). Arginase: A Critical Regulator of Nitric Oxide Synthesis and Vascular Function. Clin. Exp. Pharmacol. Physiol..

[77] Sin Y.Y., Baron G., Schulze A., Funk C.D. (2015). Arginase-1 Deficiency. J. Mol. Med..

[78] Man M.-Q., Wakefield J.S., Mauro T.M., Elias P.M. (2022). Role of Nitric Oxide in Regulating Epidermal Permeability Barrier Function. Exp. Dermatol..

[79] Sin Y.Y., Ballantyne L.L., Mukherjee K., St. Amand T., Kyriakopoulou L., Schulze A., Funk C.D. (2013). Inducible Arginase 1 Deficiency in Mice Leads to Hyperargininemia and Altered Amino Acid Metabolism. PLoS ONE.

[80] Galbiati V., Cornaghi L., Gianazza E., Potenza M.A., Donetti E., Marinovich M., Corsini E. (2018). In Vitro Assessment of Silver Nanoparticles Immunotoxicity. Food Chem. Toxicol..

[81] Yusuf A., Casey A. (2019). Surface Modification of Silver Nanoparticle (AgNP) by Liposomal Encapsulation Mitigates AgNP-Induced Inflammation. Toxicol. In Vitro.

[82] Gren S.T., Rasmussen T.B., Janciauskiene S., Håkansson K., Gerwien J.G., Grip O. (2015). A Single-Cell Gene-Expression Profile Reveals Inter-Cellular Heterogeneity within Human Monocyte Subsets. PLoS ONE.

[83] Parnsamut C., Brimson S. (2015). Effects of Silver Nanoparticles and Gold Nanoparticles on IL-2, IL-6, and TNF-α Production via MAPK Pathway in Leukemic Cell Lines. Genet. Mol. Res..

[84] Murphy A., Casey A., Byrne G., Chambers G., Howe O. (2016). Silver Nanoparticles Induce Pro-inflammatory Gene Expression and Inflammasome Activation in Human Monocytes. J. Appl. Toxicol..

[85] Ilić K., Kalčec N., Krce L., Aviani I., Turčić P., Pavičić I., Vinković Vrček I. (2022). Toxicity of Nanomixtures to Human Macrophages: Joint Action of Silver and Polystyrene Nanoparticles. Chem. Biol. Interact..

[86] Alqahtani S., Xia L., Shannahan J.H. (2022). Enhanced Silver Nanoparticle-Induced Pulmonary Inflammation in a Metabolic Syndrome Mouse Model and Resolvin D1 Treatment. Part. Fibre Toxicol..

[87] http://www.revistagastroenterologiamexico.org/es-expresion-interleucina-il-10-con-funcionarticulo-X0375090611243237.

[88] Li K., Gong Q., Lu B., Huang K., Tong Y., Mutsvene T.E., Lin M., Xu Z., Lu F., Li X. (2023). Anti-Inflammatory and Antioxidative Effects of Gallic Acid on Experimental Dry Eye: In Vitro and in Vivo Studies. Eye Vis..

[89] Lu Q.Y., Ma J.Q., Duan Y.Y., Sun Y., Yu S., Li B., Zhang G.M. (2019). Carthamin Yellow Protects the Heart Against Ischemia/Reperfusion Injury with Reduced Reactive Oxygen Species Release and Inflammatory Response. J. Cardiovasc. Pharmacol..

[90] Wu H., Lin T., Chen Y., Chen F., Zhang S., Pang H., Huang L., Yu C., Wang G., Wu C. (2023). Ethanol Extract of Rosa Laevigata Michx. Fruit Inhibits Inflammatory Responses through NF-ΚB/MAPK Signaling Pathways via AMPK Activation in RAW 264.7 Macrophages. Molecules.

[91] Thapa R., Afzal O., Alfawaz Altamimi A.S., Goyal A., Almalki W.H., Alzarea S.I., Kazmi I., Jakhmola V., Singh S.K., Dua K. (2023). Galangin as an Inflammatory Response Modulator: An Updated Overview and Therapeutic Potential. Chem. Biol. Interact..

[92] Shi Y., Zhang H., Li S., Xin D., Li S., Yan B., Wang S., Liu C. (2023). Procyanidin Improves Experimental Colitis by Regulating Macrophage Polarization. Biomed. Pharmacother..

[93] Kwon E.-Y., Choi M.-S. (2019). Dietary Eriodictyol Alleviates Adiposity, Hepatic Steatosis, Insulin Resistance, and Inflammation in Diet-Induced Obese Mice. Int. J. Mol. Sci..

[94] Rasul A., Bao R., Malhi M., Zhao B., Tsuji I., Li J., Li X. (2013). Induction of apoptosis by costunolide in bladder cancer cells is mediated through ROS generation and mitochondrial dysfunction. Molecules.

[95] Kummrow A., Frankowski M., Bock N., Werner C., Dziekan T., Neukammer J. (2013). Quantitative assessment of cell viability based on flow cytometry and microscopy. Cytom. Part A J. Int. Soc. Anal. Cytol..

[96] Romeo S., Sannino A., Scarfì M.R., Vernier P.T., Cadossi R., Gehl J., Zeni O. (2018). ESOPE-Equivalent Pulsing Protocols for Calcium Electroporation: An In Vitro Optimization Study on 2 Cancer Cell Models. Technol. Cancer Res. Treat..

[97] De Leonardis F., Barile S.N., Cianci C., Pisano I., Merla G., Pappalettera G., Casavola C., Pappalettere C. (2023). In Vitro Effects of Low-energy Ultrasound Treatment on Healthy CD3/CD8+ Lymphocytes, Red blood cells, Acute Myeloid leukemia cells, and Jurkat cell line. J. Cancer.

