# Supplementary material for: Monocyte (THP-1) Response to Silver Nanoparticles Synthesized with Rumex hymenosepalus Root Extract
Source: Nanomaterials (Basel). 2024 Jan 2;14(1):106. doi: 10.3390/nano14010106 (PMC10780692; doi:10.3390/nano14010106)
Supplement: Supplementary file 1 [file nanomaterials-14-00106-s001.zip › nanomaterials-2783281-supplementary.pdf]

# Supplementary Material

Table S1. Analysis of the global metabolite profile of the *Rumex hymenosepalus* root extract by UPLC-qTOF in negative ionization mode yielded 332 pre-identified compounds, of which 89 met the established identification criteria.

| No. | Retention time (min) | Mass (m/z) | Molecular formula | DB diff (ppm) | Name | Chemical structure |
|---|---|---|---|---|---|---|
| 1 | 1.3071 | 290.0917 | $C_{14}H_{17}N_2O_6$- | 1.1841 | 4-Carboxy-2-(tyrosylamino)butanoate | 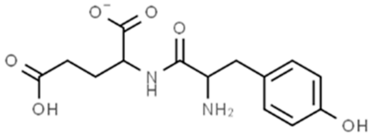 |
| 2 | 1.3071 | 404.1066 | $C_{15}H_{25}N_3O_9S$ | 15.9205 | 2-amino-4-({2-[(1-carboxy-1-hydroxy-2-methylpropan-2-yl)sulfanyl]-1-[(carboxymethyl)-C-hydroxycarbonimidoyl]ethyl}-C-hydroxycarbonimidoyl)butanoic acid | 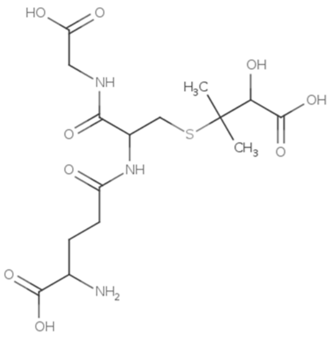 |
| 3 | 1.3577 | 353.0766 | $C_{11}H_{16}O_{10}$ | 13.2929 | D-Erythroascorbic acid 1'-a-D-glucoside | 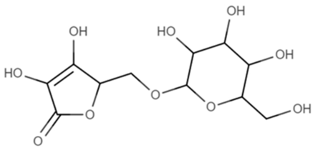 |
| 4 | 1.3577 | 1123.3120 | $C_{26}H_{28}O_{12}$ | -16.8823 | Sesaminol glucoside | 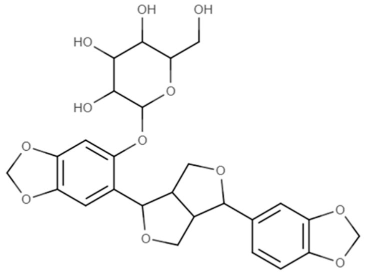 |
| **5** | 1.4084 | 111.0112 | $C_5H_6O_4$ | 18.4584 | 2,5-Dioxopentanoate | 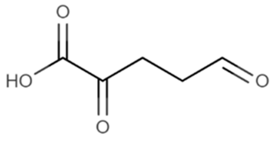 |

| | | | | | | |
|---|---|---|---|---|---|---|
| 6 | 1.6163 | 429.0392 | $C_{19}H_{20}O_7S$ | -6.1624 | {[(3E)-4-{2,6-dihydroxy-4-[(E)-2-(4-hydroxyphenyl)ethenyl]phenyl}-2-methylbut-3-en-1-yl]oxy}sulfonic acid | 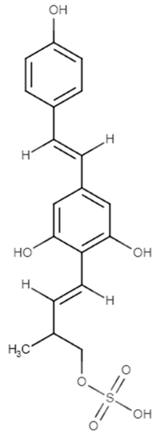 |
| **7** | 1.6697 | 331.0702 | $C_{13}H_{16}O_{10}$ | 9.3147 | 2-Galloylglucose | 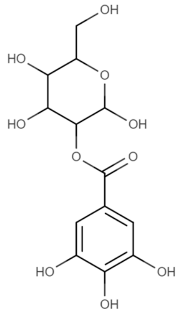 |
| 8 | 1.7177 | 379.0361 | $C_{12}H_{17}N_4O_4PS$ | -11.8888 | Thiamine monophosphate | 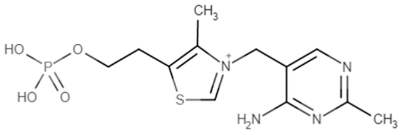 |
| **9** | 1.9763 | 125.0272 | $C_6H_8O_4$ | 19.3183 | 3-Methylglutaconic acid | 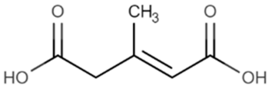 |
| **10** | 1.9763 | 169.0168 | $C_7H_6O_5$ | 15.2187 | Gallic acid | 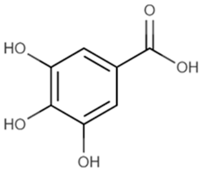 |
| 11 | 2.0270 | 739.1910 | $C_{36}H_{36}O_{17}$ | 4.1446 | Kaempferol 3-[6'''-p-coumarylglucosyl-(1->2)-rhamnoside] | 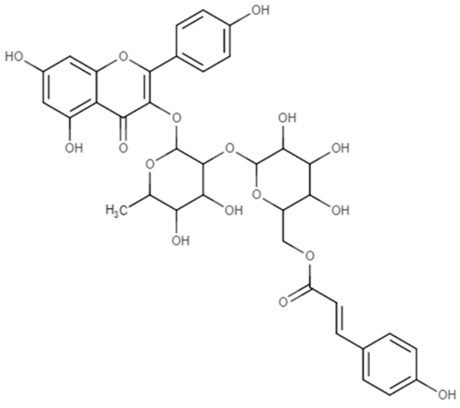 |

| | | | | | | |
|---|---|---|---|---|---|---|
| 12 | 2.1056 | 425.0417 | $C_{18}H_{16}N_2O_8$ | 6.3468 | dopaxanthin quinone | 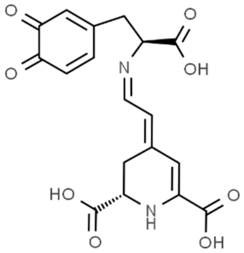 |
| 13 | 2.1056 | 655.0999 | $C_{28}H_{26}O_{17}$ | 12.9428 | 3'-(2'',6''-Digalloylglucosyl)-phloroacetophenone | 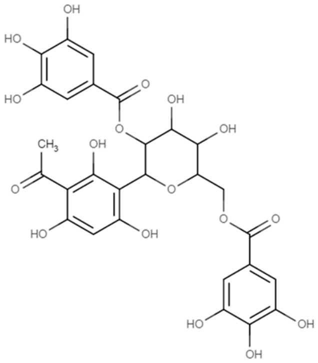 |
| **14** | 2.4656 | 255.0527 | $C_{10}H_{10}O_5$ | 7.9841 | 3-(4-hydroxy-3-methoxyphenyl)oxirane-2-carboxylic acid | 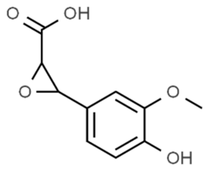 |
| 15 | 2.7242 | 197.0489 | $C_9H_{10}O_5$ | 16.9560 | Ethyl gallate | 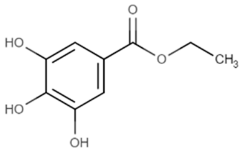 |
| 16 | 2.7749 | 641.1168 | $C_{28}H_{28}O_{16}$ | 7.0869 | 6-{4-[6-(3,4-dihydroxy-6-methyl-5-oxooxan-2-yl)-5,7-dihydroxy-4-oxo-4H-chromen-2-yl]-2-methoxyphenoxy}-3,4,5-trihydroxyoxane-2-carboxylic acid | 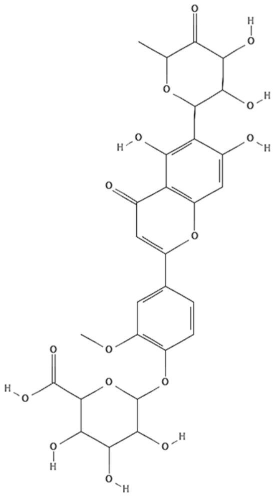 |
| 17 | 3.1627 | 359.1006 | $C_8H_8N_2O_3$ | 2.4219 | Nicotinuric acid | 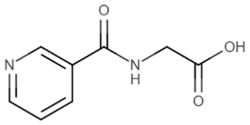 |

| | | | | | | |
|---|---|---|---|---|---|---|
| 18 | 3.2134 | 425.0910 | $C_{20}H_{20}O_9$ | 13.9599 | Cassiaside | 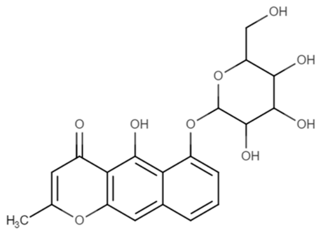 |
| **19** | 3.2134 | 577.1360 | $C_{30}H_{26}O_{12}$ | 1.5416 | 2-{[5,7-dihydroxy-2-(4-hydroxyphenyl)-4-oxo-4H-chromen-3-yl]oxy}-4,5-dihydroxy-6-methyloxan-3-yl (2E)-3-(4-hydroxyphenyl)prop-2-enoate | 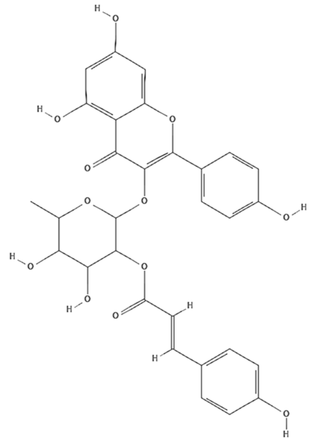 |
| **20** | 3.2642 | 407.0666 | $C_{16}H_{18}O_{11}$ | 18.2154 | 6-[4-(2-carboxyeth-1-en-1-yl)-5-hydroxy-2-methoxyphenoxy]-3,4,5-trihydroxyoxane-2-carboxylic acid | 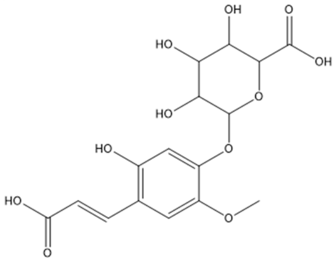 |
| 21 | 3.3428 | 239.0599 | $C_{10}H_{10}O_4$ | 19.6399 | (-)-trans-3,4-Dihydro-4,8-dihydroxy-3-methyl-1H-2-benzopyran-1-one | 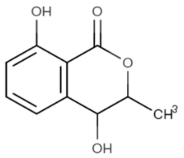 |
| 22 | 3.3428 | 727.1432 | $C_{37}H_{30}O_{17}$ | 17.1185 | Epigallocatechin-(4beta->8)-epicatechin-3-O-gallate ester | 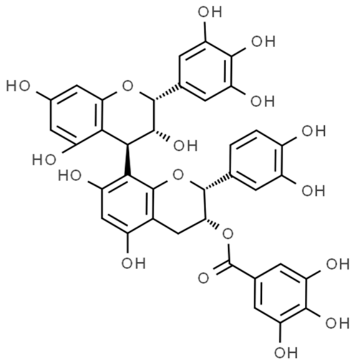 |

| | | | | | | |
|---|---|---|---|---|---|---|
| **23** | 3.3428 | 1151.2337 | $C_{60}H_{48}O_{24}$ | 10.8713 | Pavetannin C1 | 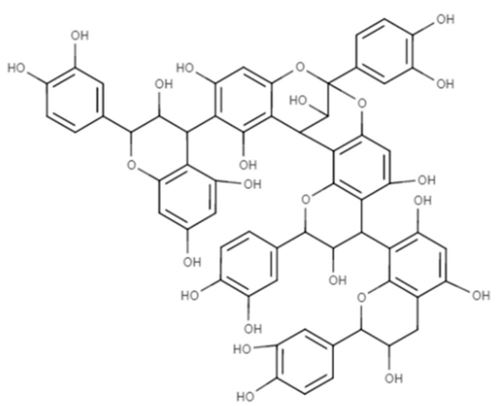 |
| 24 | 3.3934 | 205.0737 | $C_{12}H_{12}N_2$ | 5.4815 | Harmalan | 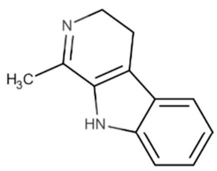 |
| 25 | 3.5227 | 393.1219 | $C_{19}H_{22}O_9$ | 7.0248 | 2'-Oxoaloesol 7-glucoside | 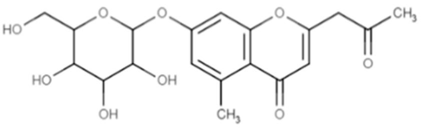 |
| 26 | 3.5227 | 1015.2069 | $C_{44}H_{50}O_{25}$ | -5.9159 | Kaempferol 3-O-sinapoyl-sophoroside 7-O-glucoside | 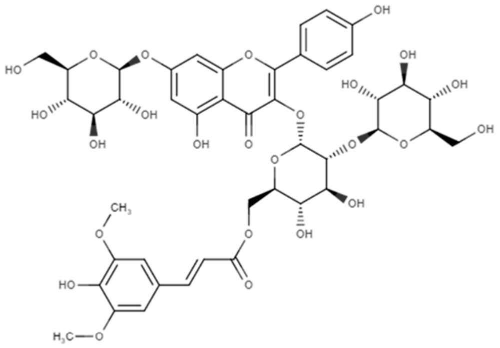 |
| 27 | 3.6520 | 739.1884 | $C_{36}H_{36}O_{17}$ | 0.5871 | Kaempferol 2G-coumaroylrutinoside | 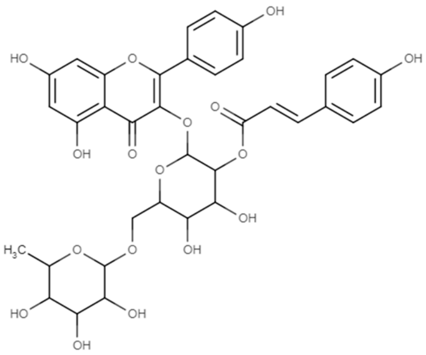 |
| 28 | 3.7027 | 317.0704 | $C_{16}H_{14}O_7$ | 11.6586 | (2R)-2,7-Dihydroxy-2-[(6-hydroxy-4-oxo-4H-pyran-2-yl)methyl]-5-methyl-2,3-dihydro-4H-chromen-4-one | 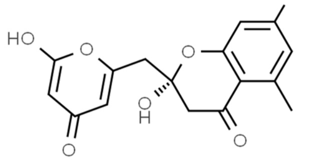 |

| | | | | | | |
|---|---|---|---|---|---|---|
| **29** | 3.7813 | 289.0740 | $C_{15}H_{14}O_6$ | 7.6017 | 6-[(E)-2-(2H-1,3-benzodioxol-5-yl)ethenyl]-5-hydroxy-4-methoxy-5,6-dihydro-2H-pyran-2-one (Methysticin-ol) | 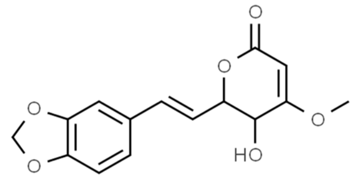 |
| 30 | 3.7813 | 449.1102 | $C_{21}H_{24}O_{12}$ | 2.7278 | 8-[3,4,5-trihydroxy-6-(hydroxymethyl)oxan-2-yl]-2-(2,4,5-trihydroxyphenyl)-3,4-dihydro-2H-1-benzopyran-3,5,7-triol | 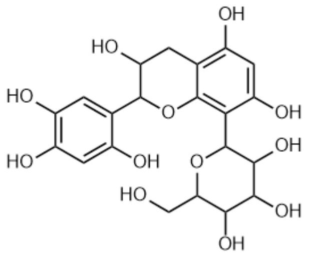 |
| **31** | 3.7813 | 891.1967 | $C_{43}H_{42}O_{22}$ | -2.4359 | Carthamin | 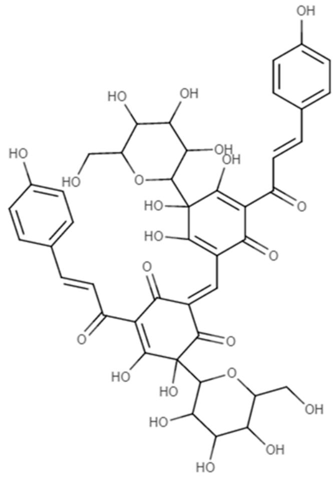 |
| **32** | 3.8827 | 865.1973 | $C_{45}H_{38}O_{18}$ | -1.4581 | Procyanidin C1 | 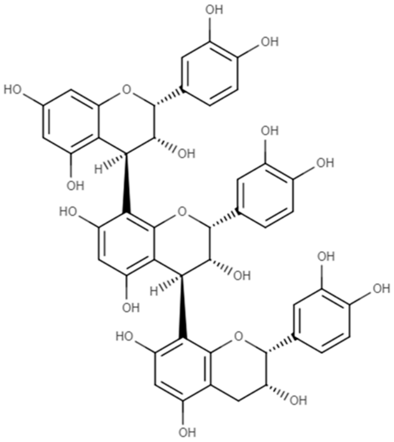 |

| | | | | | | |
|---|---|---|---|---|---|---|
| 33 | 3.9613 | 451.1085 | $C_{21}H_{26}O_{10}S$ | 3.6211 | [(1-{10-butanoyl-5,7-dihydroxy-8,8-dimethyl-2-oxo-2H,6H,7H,8H-pyrano[3,2-g]chromen-4-yl}propan-2-yl)oxy]sulfonic acid | 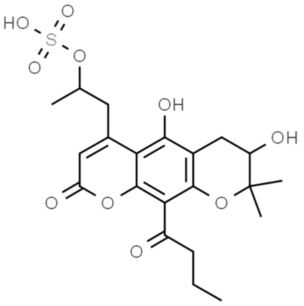 |
| **34** | 3.9613 | 577.1295 | $C_{30}H_{28}O_{13}$ | -9.5345 | Eriodictyol 7-(6-trans-p-coumaroylglucoside) | 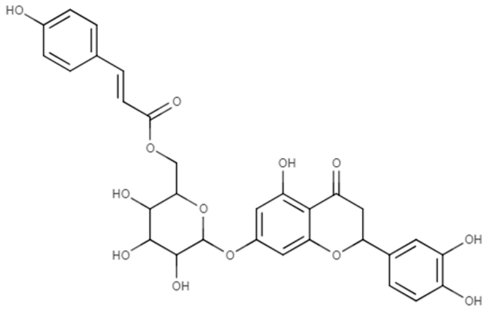 |
| 35 | 4.012 | 299.0601 | $C_{16}H_{14}O_{7}$ | 12.6296 | 3,5,6,7-tetrahydroxy-2-(4-hydroxy-3-methoxyphenyl)-8aH-chromen-8a-yl | 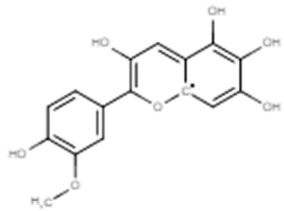 |
| 36 | 4.0906 | 693.1835 | $C_{16}H_{17}N_{3}O_{4}S$ | 4.1370 | Cephalexin | 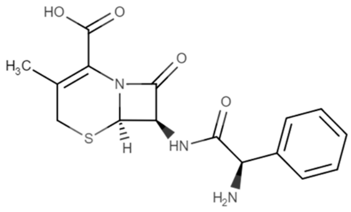 |
| 37 | 4.1413 | 467.09066 | $C_{18}H_{24}O_{12}$ | -2.7719 | Licoagroside B | 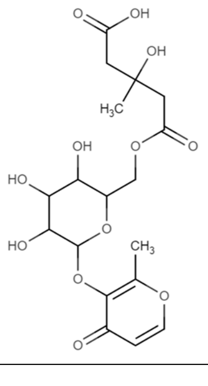 |

| | | | | | | |
|---|---|---|---|---|---|---|
| 38 | 4.19 | 745.18 | $C_{36}H_{36}O_{16}$ | 5.4548 | 4-[2-(3,4-dihydroxyphenyl)-3,5,7-trihydroxy-3,4-dihydro-2H-1-benzopyran-8-yl]-2-(3-hydroxyphenyl)-6-[3,4,5-trihydroxy-6-(hydroxymethyl)oxan-2-yl]-3,4-dihydro-2H-1-benzopyran-3,5,7-triol | 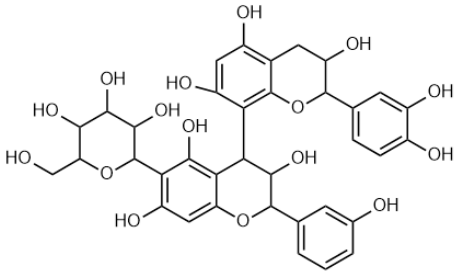 |
| 39 | 4.3213 | 969.2088 | $C_{43}H_{48}O_{23}$ | 1.6312 | Isovitexin 2''-(6'''-feruloylglucoside) 4'-glucoside | 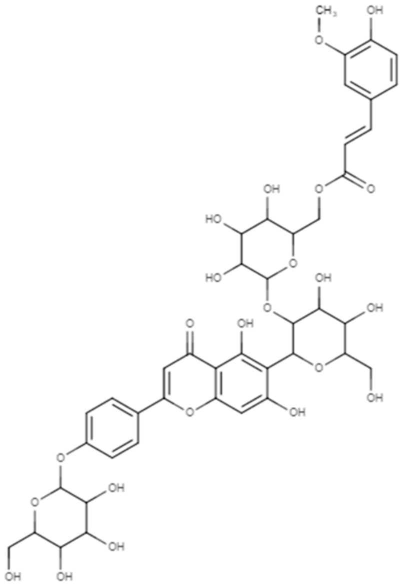 |
| **40** | 4.3999 | 289.0732 | $C_{15}H_{14}O_{6}$ | 4.8138 | Marshrin | 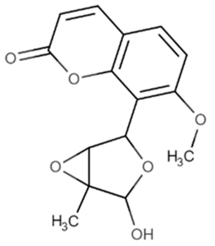 |
| 41 | 4.45 | 483.10 | $C_{20}H_{22}O_{9}S$ | -2.1114 | {4,8-dihydroxy-17-methoxy-10-oxo-2-oxatricyclo[13.2.2.1Â $^{3}$,â ·]icosa-1(17),3,5,7(20),15,18-hexaen-14-yl}oxidanesulfonic acid | 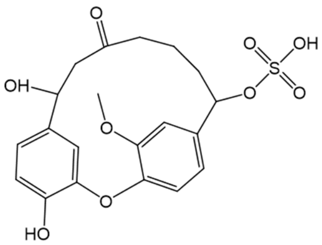 |

| | | | | | | |
|---|---|---|---|---|---|---|
| 42 | 4.45 | 603.14 | $C_{28}H_{30}O_{16}$ | 0.9553 | 3,4,5-trihydroxy-6-(4-{5-hydroxy-7-methoxy-4-oxo-8-[3,4,5-trihydroxy-6-(hydroxymethyl)oxan-2-yl]-4H-chromen-2-yl}phenoxy)oxane-2-carboxylic acid | 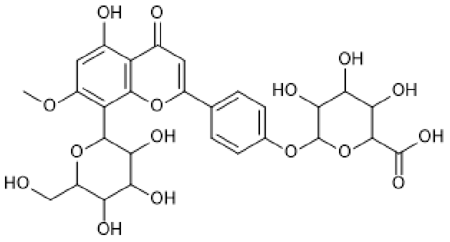 |
| **43** | 4.5013 | 1169.2134 | $C_{59}H_{46}O_{26}$ | -6.0135 | Epicatechin 3-O-gallate-(4beta->6)-epicatechin 3-O-gallate-(4beta->8)-catechin | 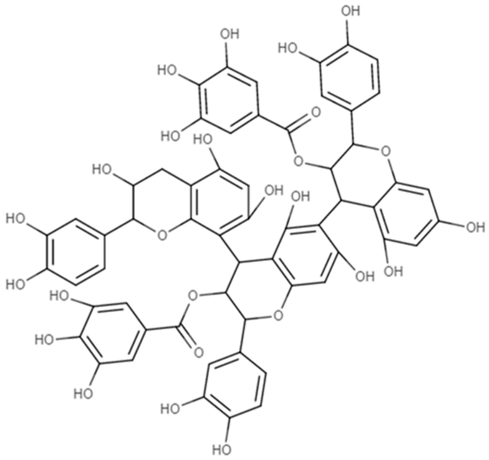 |
| 44 | 4.5013 | 711.1375 | $C_{29}H_{30}O_{18}$ | -5.9282 | (3''-Apiosyl-6''-malonyl)astragalin | 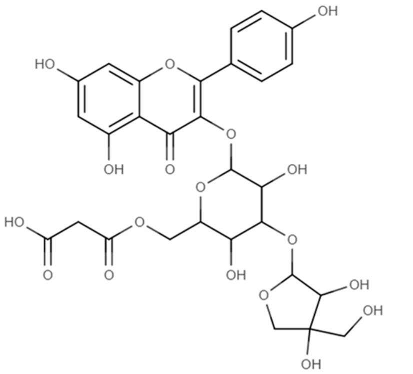 |
| 45 | 4.5799 | 1043.2039 | $C_{23}H_{22}O_{14}$ | 8.8775 | Spinatoside | 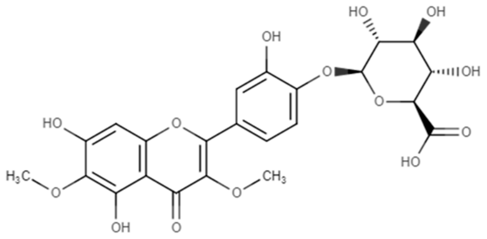 |
| **46** | 4.6306 | 243.0665 | $C_{14}H_{14}O_5$ | 0.9104 | 9,10-dihydroxy-8,8-dimethyl-2H,8H,9H,10H-pyrano[2,3-h]chromen-2-one | 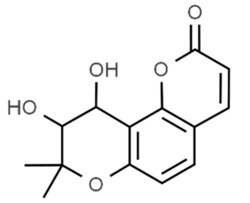 |

| | | | | | | |
|---|---|---|---|---|---|---|
| **47** | 4.6306 | 405.1191 | $C_{20}H_{24}O_{10}$ | 0.0938 | Rutarin | 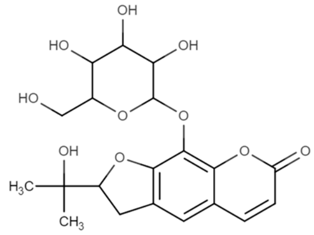 |
| 48 | 4.7599 | 693.1865 | $C_{35}H_{34}O_{15}$ | 5.7239 | 6-{2-[6-carboxy-5-(2,4-dihydroxyphenyl)-3-methylcyclohex-2-en-1-yl]-4-[(2E)-3-(2,4-dihydroxyphenyl)prop-2-enoyl]-3-hydroxyphenoxy}-3,4,5-trihydroxyoxane-2-carboxylic acid | 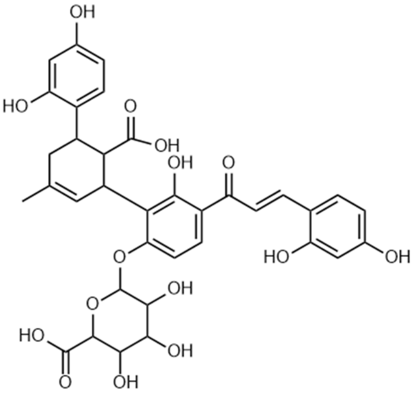 |
| **49** | 4.8106 | 227.0733 | $C_{14}H_{14}O_4$ | 7.8986 | Aegelinol | 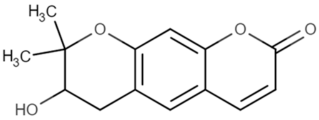 |
| 50 | 4.9399 | 457.0818 | $C_{22}H_{20}O_{12}$ | 8.6675 | 6-[3-(5,7-dihydroxy-4-oxo-4H-chromen-2-yl)-5-methoxyphenoxy]-3,4,5-trihydroxyoxane-2-carboxylic acid | 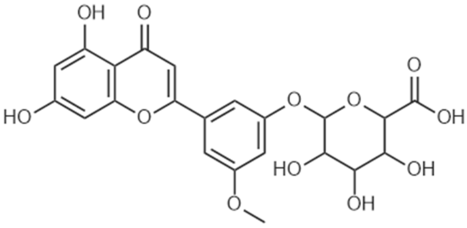 |
| 51 | 5.0185 | 845.1925 | $C_{41}H_{36}O_{17}$ | -1.2135 | 5,7-Dihydroxy-2-(4-hydroxy-3-methoxyphenyl)-4-oxo-4H-chromen-3-yl 6-O-[(2E)-3-(4-hydroxy-3-methoxyphenyl)-2-propenoyl]-3-O-[(2E)-3-(4-hydroxyphenyl)-2-propenoyl]-beta-D-glucopyranoside | 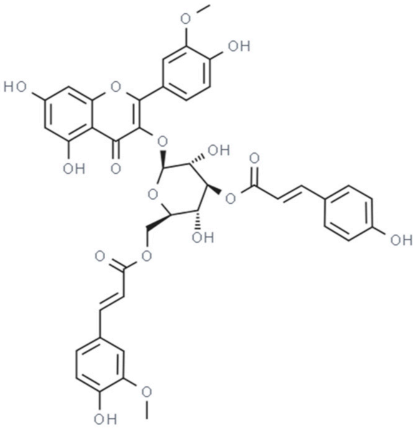 |
| 52 | 5.4291 | 197.04862 | $C_9H_{10}O_5$ | 15.4930 | 2-hydroxy-2-(2-hydroxy-4-methoxyphenyl)acetic acid | 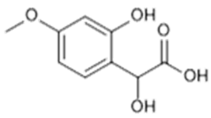 |

| | | | | | | |
|---|---|---|---|---|---|---|
| **53** | 5.5584 | 442.0900 | $C_{22}H_{18}O_{10}$ | -6.8474 | (-)-Epicatechin 3-O-gallate | 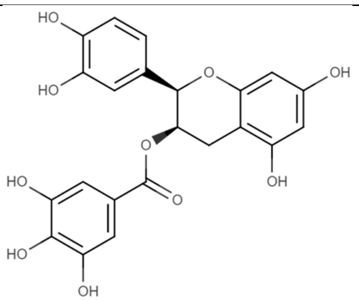 |
| 54 | 5.6371 | 557.1310 | $C_{26}H_{24}O_{11}$ | 1.8627 | 3,4,5-trihydroxy-6-(2-{5-hydroxy-8,8-dimethyl-2-oxo-2H,8H-pyrano[2,3-f]chromen-4-yl}phenoxy)oxane-2-carboxylic acid | 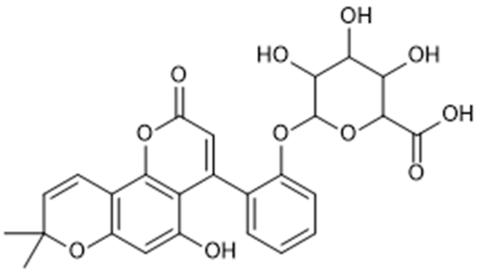 |
| 55 | 5.7385 | 865.1644 | $C_{44}H_{34}O_{19}$ | 2.6490 | Epiafzelechin-(4beta->6)-epicatechin 3,3'-digallate | 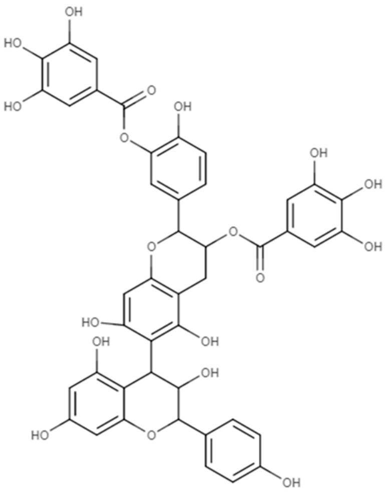 |
| 56 | 5.9464 | 541.1387 | $C_{27}H_{28}O_{13}$ | 6.3405 | Apimaysin | 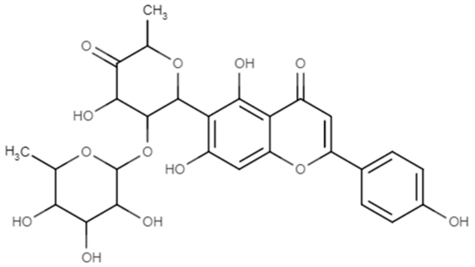 |

| | | | | | | |
|---|---|---|---|---|---|---|
| 57 | 6.1263 | 577.1100 | $C_{27}H_{26}O_{12}$ | -3.4400 | Resveratrol 4'-(2-galloylglucoside) | 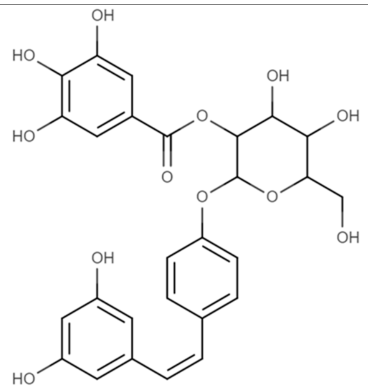 |
| **58** | 6.1770 | 729.1457 | $C_{35}H_{34}O_{15}$ | -19.4347 | 6-{4-[(1E)-3-{3-[6-carboxy-5-(2,4-dihydroxyphenyl)-3-methylcyclohex-2-en-1-yl]-2,4-dihydroxyphenyl}-3-oxoprop-1-en-1-yl]-3-hydroxyphenoxy}-3,4,5-trihydroxyoxane-2-carboxylic acid | 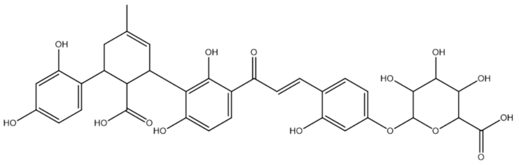 |
| 59 | 6.2556 | 243.0701 | $C_{14}H_{14}O_5$ | 14.5950 | 6-[2-(2H-1,3-benzodioxol-5-yl)ethyl]-4-hydroxy-5,6-dihydro-2H-pyran-2-one | 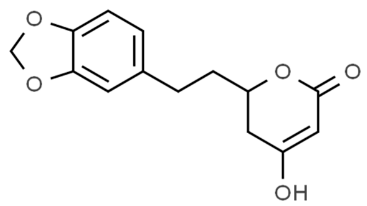 |
| 60 | 6.5650 | 407.0814 | $C_{22}H_{18}O_9$ | 9.8032 | (-)-Epiafzelechin 3-gallate | 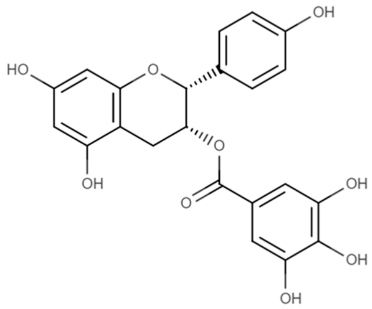 |
| **61** | 6.5650 | 881.1532 | $C_{44}H_{34}O_{20}$ | -4.4121 | Epiafzelechin 3-O-gallate-(4beta->6)-epigallocatechin 3-O-gallate | 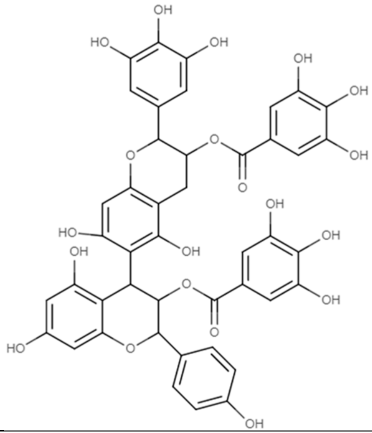 |

| | | | | | | |
|---|---|---|---|---|---|---|
| 62 | 6.7955 | 683.1444 | $C_{36}H_{30}O_{15}$ | 5.4068 | 6-{3-[8-(5,7-dihydroxy-4-oxo-2-phenyl-3,4-dihydro-2H-1-benzopyran-3-yl)-5,7-dihydroxy-4-oxo-3,4-dihydro-2H-1-benzopyran-2-yl]phenoxy}-3,4,5-trihydroxyoxane-2-carboxylic acid | 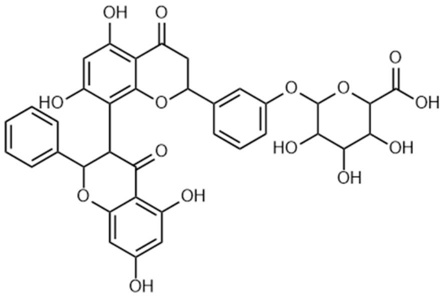 |
| 63 | 7.1048 | 469.1191 | $C_{21}H_{26}O_{10}S$ | 3.5804 | (3-{10-butanoyl-5,7-dihydroxy-8,8-dimethyl-2-oxo-2H,6H,7H,8H-pyrano[3,2-g]chromen-4-yl}propoxy)sulfonic acid | 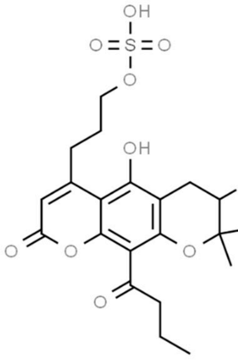 |
| 64 | 7.1048 | 865.1624 | $C_{44}H_{34}O_{19}$ | 0.3116 | Epiafzelechin-(4b->8)-epicatechin 3,3'-digallate | 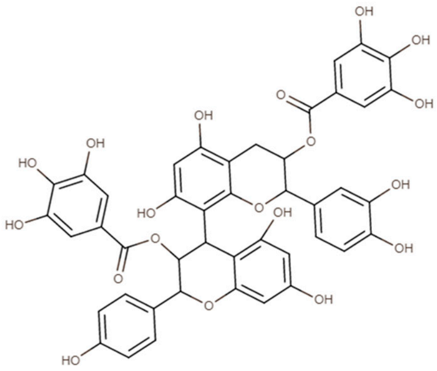 |
| **65** | 7.4927 | 227.0744 | $C_{14}H_{12}O_3$ | 13.1308 | 5,6-Dihydro-5-hydroxy-6-methyl-2H-pyran-2-one (5,6-dehydrokawain) | 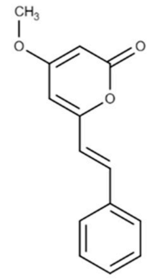 |
| 66 | 7.5940 | 317.0698 | $C_{16}H_{16}O_8$ | 9.4074 | 3-O-Caffeoylshikimic acid | 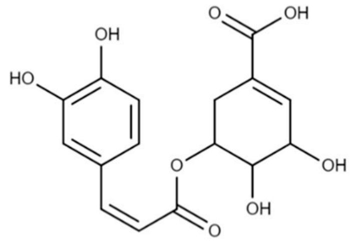 |

| | | | | | | |
|---|---|---|---|---|---|---|
| **67** | 7.7233 | 377.1283 | $C_{19}H_{24}O_9$ | 10.4098 | Aloesol 7-glucoside | 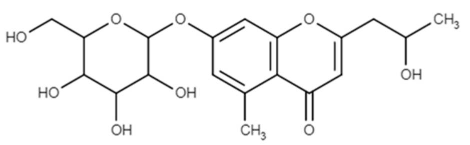 |
| **68** | 8.2127 | 285.0443 | $C_{12}H_{16}O_7S$ | 1.4553 | 5-(3-methoxyphenyl)-4-(sulfooxy)pentanoic acid | 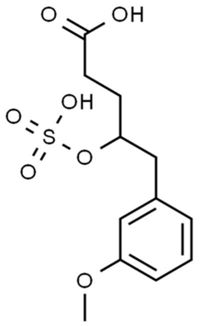 |
| **69** | 8.2127 | 301.0390 | $C_{15}H_{10}O_7$ | 12.0165 | Quercetin | 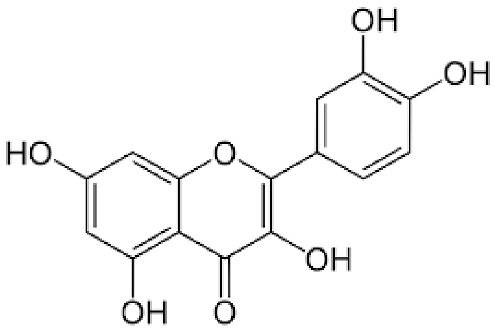 |
| 70 | 8.3419 | 253.05381 | $C_{15}H_{12}O_5$ | 11.6772 | 5,6,7-trihydroxy-3-phenyl-3,4-dihydro-2H-1-benzopyran-4-one | 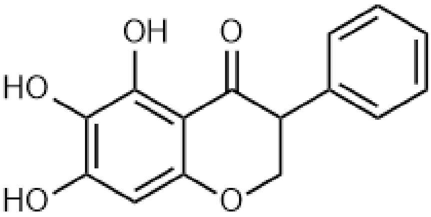 |
| 71 | 8.3419 | 617.1325 | $C_{31}H_{24}O_{11}$ | 4.3430 | 3-[5,7-dihydroxy-2-(4-hydroxy-3-methoxyphenyl)-4-oxo-3,4-dihydro-2H-1-benzopyran-8-yl]-5,7-dihydroxy-2-(4-hydroxyphenyl)-3,4-dihydro-2H-1-benzopyran-4-one | 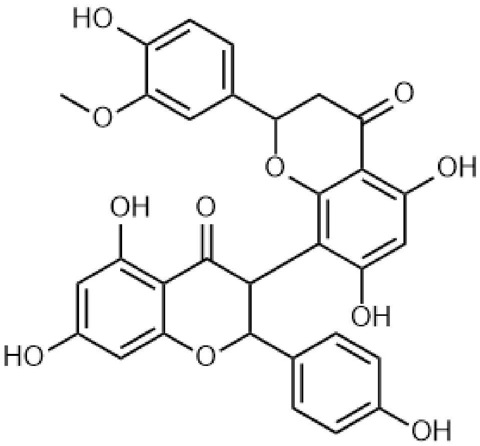 |

| | | | | | | |
|---|---|---|---|---|---|---|
| 72 | 8.4712 | 255.0696 | $C_{15}H_{14}O_5$ | 12.0352 | 2-phenyl-3,4-dihydro-2H-1-benzopyran-3,4,5,7-tetrol | 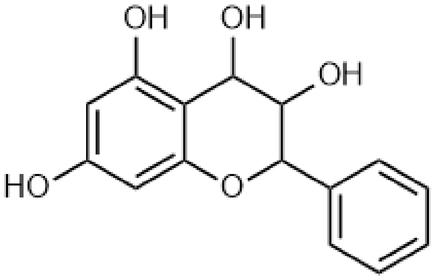 |
| 73 | 8.7299 | 345.0654 | $C_{17}H_{16}O_9$ | 10.5744 | Xanthotoxol glucoside | 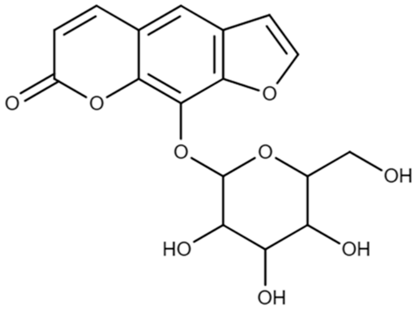 |
| 74 | 9.1406 | 269.0491 | $C_{15}H_{10}O_5$ | 13.3045 | 6-Hydroxy-2-(6-oxo-1-oxaspiro[2.5]octa-4,7-dien-2-yl)-1-benzofuran-3(2H)-one | 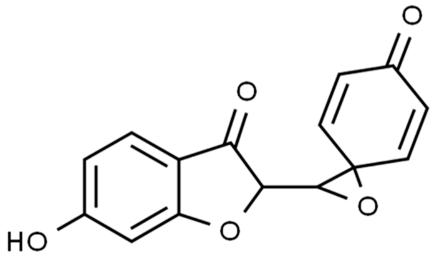 |
| 75 | 9.2192 | 253.0544 | $C_{15}H_{12}O_5$ | 13.7615 | Naringenin | 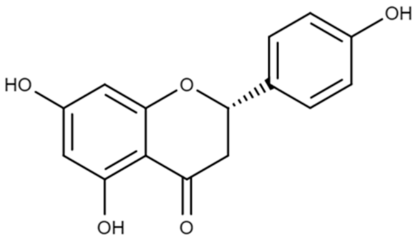 |
| 76 | 9.2192 | 271.0648 | $C_{15}H_{12}O_5$ | 13.1557 | 3,5,7-trihydroxy-2-(3-hydroxyphenyl)-5H-chromen-5-yl | 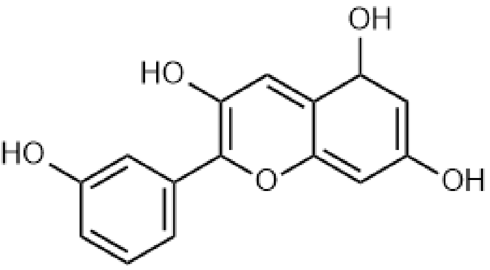 |
| 77 | 9.7591 | 285.0448 | $C_{15}H_{12}O_7$ | 14.1884 | 2-(3,5-Dihydroxyphenyl)-5,6,7-trihydroxy-2,3-dihydro-4H-chromen-4-one | 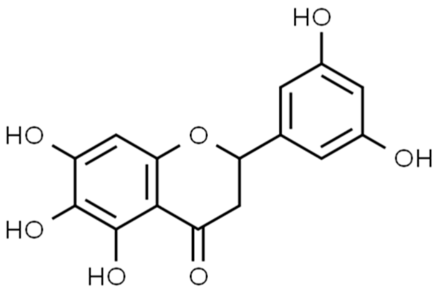 |

| | | | | | | |
|---|---|---|---|---|---|---|
| 78 | 10.8949 | 297.0436 | $C_{16}H_{10}O_6$ | 10.6796 | 3,8-Dihydroxy-1-methylanthraquinone-2-carboxylic acid | 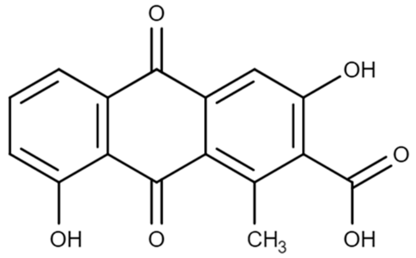 |
| 79 | 11.2041 | 457.1335 | $C_{25}H_{24}O_7$ | 15.2724 | Artonin J | 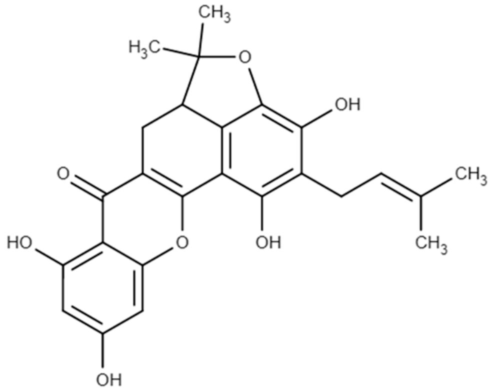 |
| 80 | 11.5134 | 365.0696 | $C_{14}H_{12}N_2O_7$-2 | 18.1460 | 2-{[(3-Hydroxy-2-oxo-2,3-dihydro-1H-indol-3-yl)acetyl]amino}succinate | 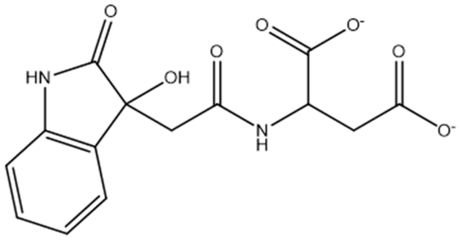 |
| 81 | 12.0535 | 255.0699 | $C_{15}H_{14}O_5$ | 13.1428 | 2-(3-Hydroxyphenyl)-1-(2,4,5-trihydroxyphenyl)-1-propanone | 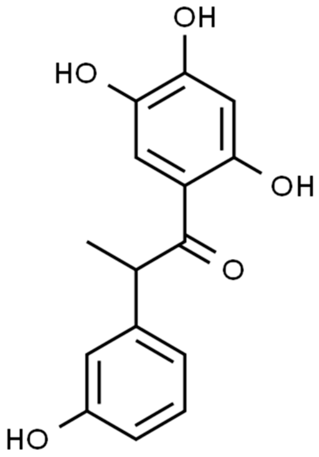 |
| **82** | 13.9092 | 269.0475 | $C_{15}H_{10}O_5$ | 7.2254 | Galangin | 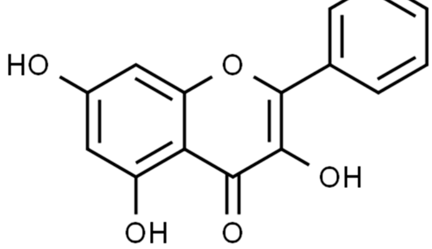 |

| | | | | | | |
|---|---|---|---|---|---|---|
| 83 | 14.5277 | 677.3793 | $C_{33}H_{58}O_{14}$ | 5.7915 | Gingerglycolipid B | 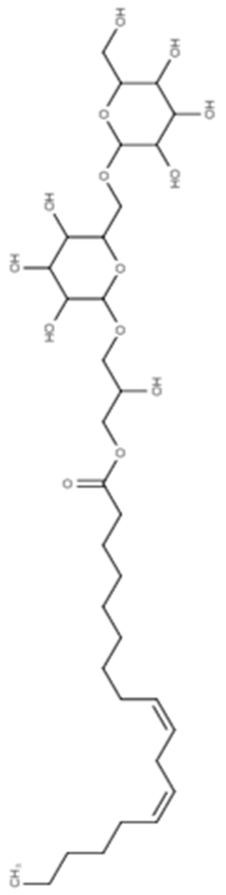 |

| | | | | | | |
|---|---|---|---|---|---|---|
| 84 | 15.6355 | 452.2823 | $C_{21}H_{44}NO_7P$ | 8.9304 | LysoPE(16:0/0:0) | 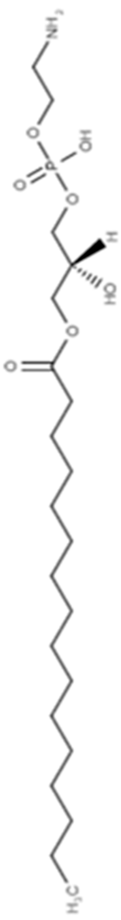 |

| | | | | | | |
|---|---|---|---|---|---|---|
| 85 | 16.2034 | 478.2977 | $C_{23}H_{46}NO_7P$ | 7.9184 | LysoPE(18:1(9Z)/0:0) | 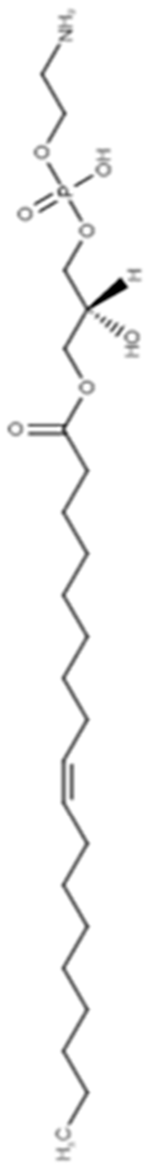 |
| 86 | 16.3327 | 431.2241 | $C_{23}H_{38}O_5$ | 9.0326 | Tylactone | 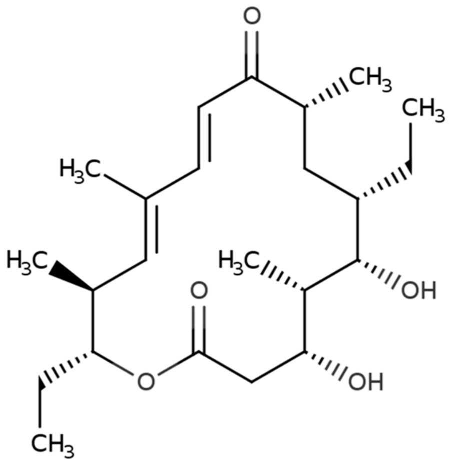 |

| | | | | | | |
|---|---|---|---|---|---|---|
| 87 | 17.8791 | 597.3085 | $C_{22}H_{44}N_6O_{10}$ | -2.8495 | Arbekacin | 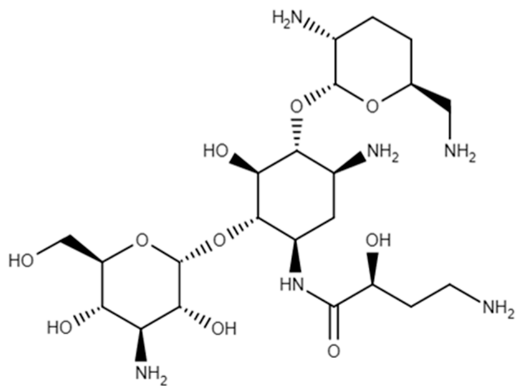 |
| **88** | 18.0591 | 433.2394 | $C_{21}H_{39}O_7P$ | 7.6162 | LysoPA(0:0/18:2(9Z, 12Z)) | 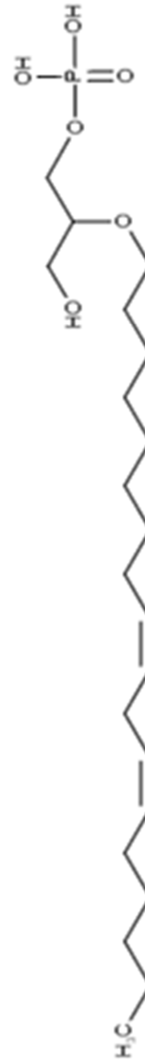 |

| | | | | | | |
|---|---|---|---|---|---|---|
| 89 | 19.7854 | 271.2325 | $C_{16}H_{32}O_3$ | 17.1867 | 16-Hydroxy hexadecanoic acid | 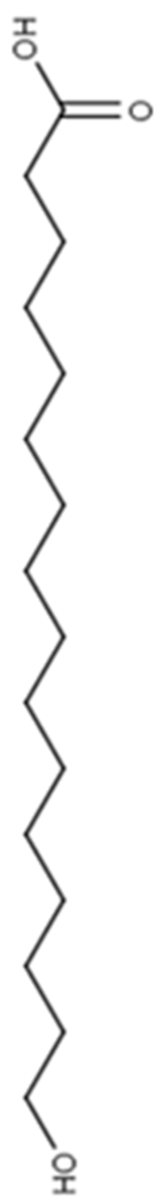 |

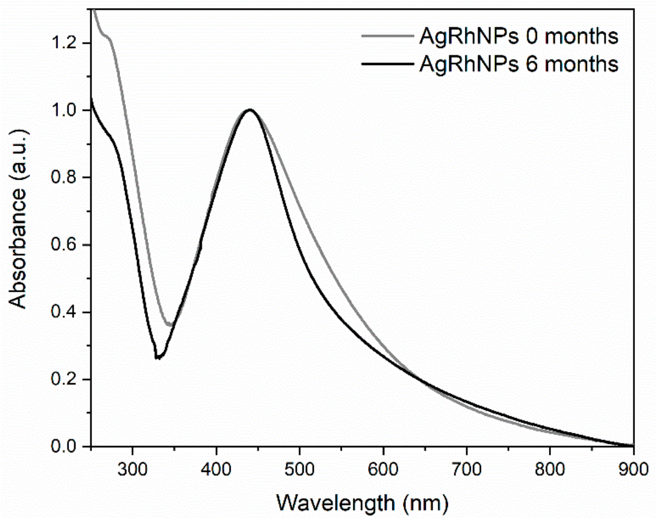


**Figure S1.** Stability Study of AgRhNPs Using UV-Vis Spectra

**Table S2.** Zeta Potential to AgRhNPs at 0 and 6 months.

| Storage Time | Zeta-potential (mV) |
|---|---|
| AgRhNPs at 0 months | -40.2 ± 5.9 |
| AgRhNPs at 6 months | -26.5 ± 5.2 |

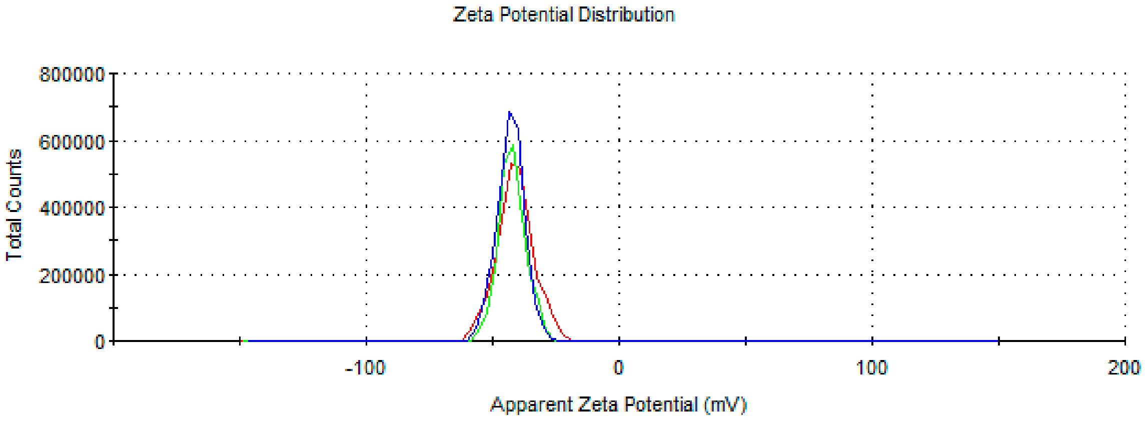


**Figure S2.** Zeta-potential of newly synthesized AgRhNPs

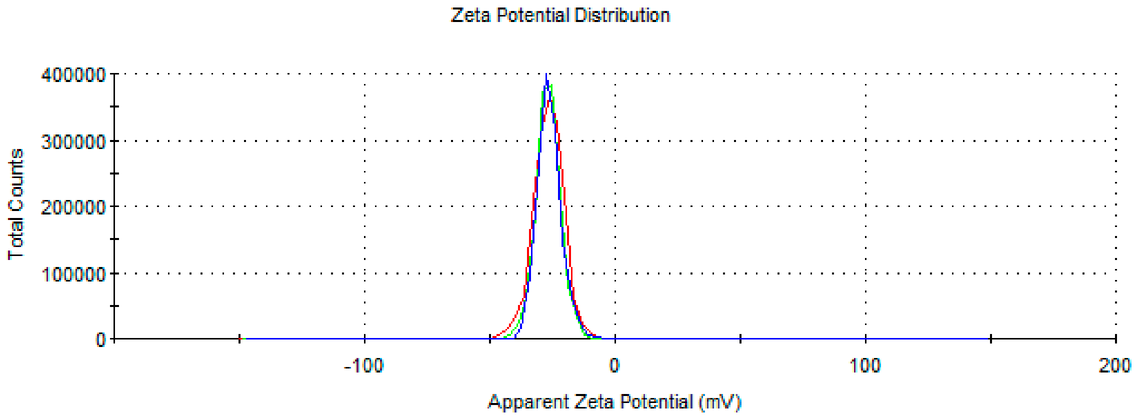


**Figure S3.** Zeta-potential of AgRhNPs at 6 months of storaged

## Cell viability assay using calcein-AM by flow cytometry

The viability assays present results as a relative comparison between 100% viability or even a total of 1 compared to the experimental treatments, as commonly seen in assays such as MTT. In vitro viability assays recognize that a certain percentage of untreated cells are not viable due to various processes, such as cells naturally undergoing apoptosis. Therefore, although we have referred to the control cells as "untreated cells", they certainly cannot be described as intact. These untreated control cells, like the treated cells, have been exposed to specific growth conditions and, like the treated cells, continue to undergo cell death. An advantage of viability assays performed by flow cytometry is the ability to provide results consistent with this situation. In the calcein-am assay, untreated cells may not stain and are therefore classified as non-viable cells by flow cytometry [1-4]. Finally, to avoid confusion, we have included as a supplemental figure the treatments at 6 and 12 h, which include controls as well as untreated cells. Here it's clear that a certain percentage of untreated cells appear as non-viable (cells not stained with calcein AM).

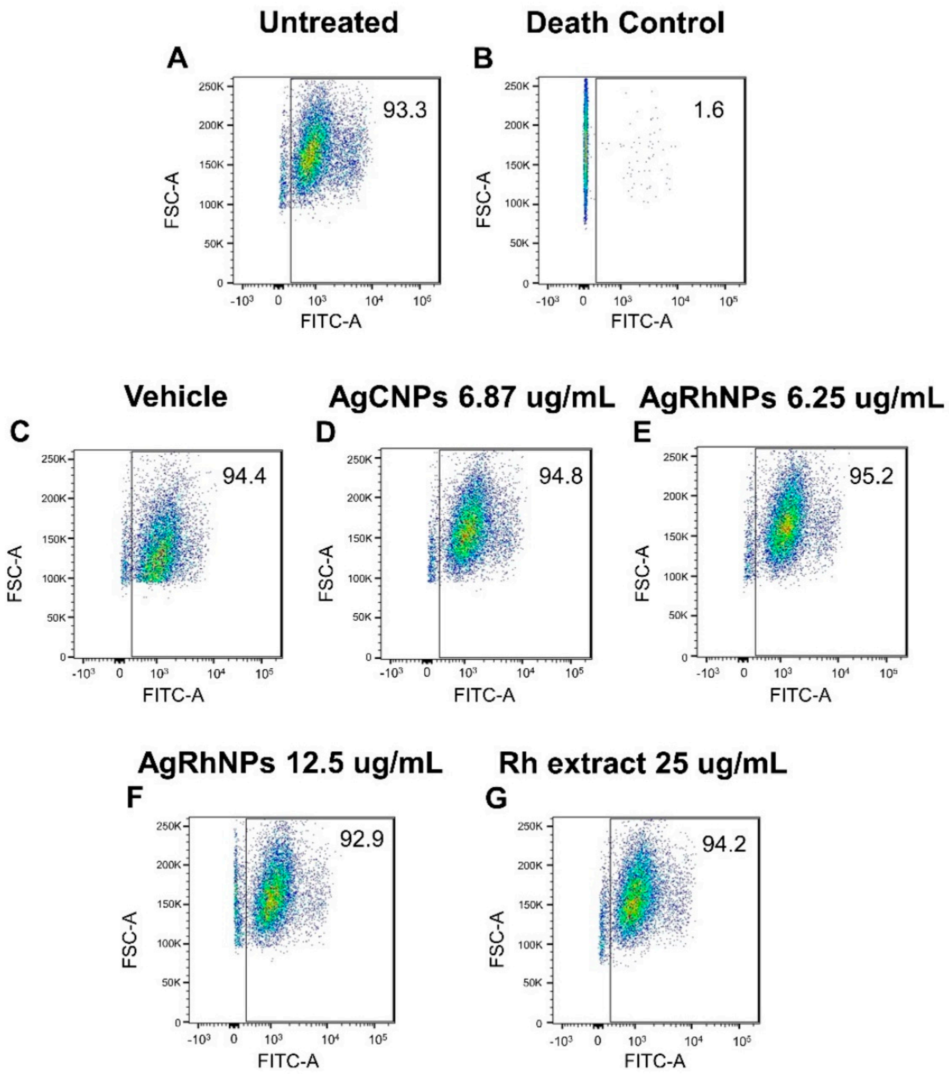


**Figure S4.** Cell viability assay using calcein-AM . Different treatments were administered to THP-1 and quantified at 6 h. For the viability assay $1 \times 10^4$ events were analyzed by flow cytometry using a BD FACS Verse flow cytometer (BD Bioscience). Data is shown as percentage of viable cells in the FITC-A-channel. Calcein-AM solution was added at 5μM.

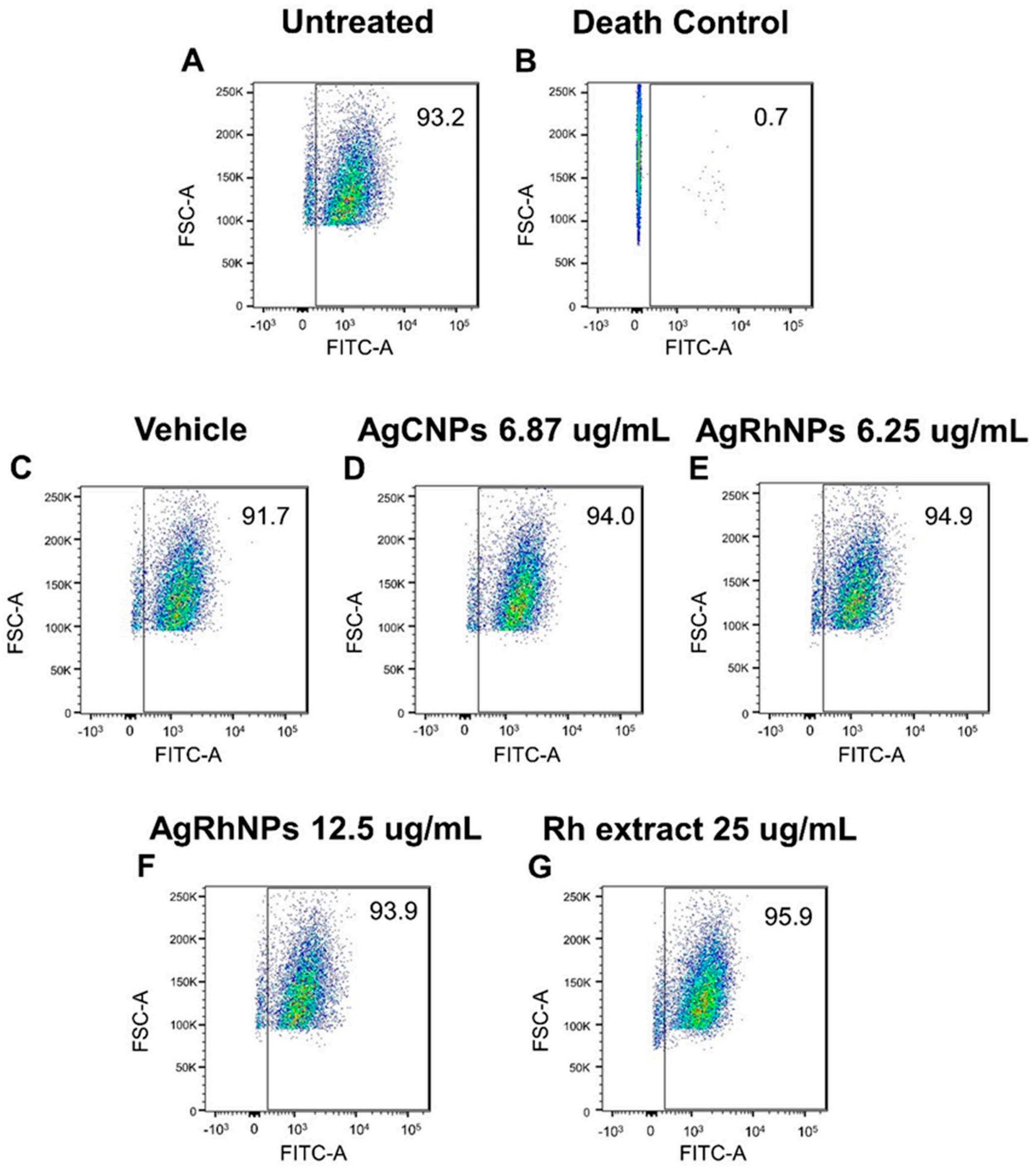


**Figure S5.** Cell viability assay using calcein-AM . Different treatments were administered to THP-1 and quantified at 12 h. For the viability assay $1 \times 10^4$ events were analyzed by flow cytometry using a BD FACS Verse flow cytometer (BD Bioscience). Data is shown as percentage of viable cells in the FITC-A-channel. Calcein-AM solution was added at 5μM.

References


1. Rasul, Azhar et al. "Induction of apoptosis by costunolide in bladder cancer cells is mediated through ROS generation and mitochondrial dysfunction." Molecules (Basel, Switzerland) vol. 18,2 1418-33. 24 Jan. 2013, doi:10.3390/molecules18021418.
2. Kummrow, A et al. "Quantitative assessment of cell viability based on flow cytometry and microscopy." Cytometry. Part A : the journal of the International Society for Analytical Cytology vol. 83,2 (2013): 197-204. doi:10.1002/cyto.a.22213.

3. Romeo, Stefania et al. “ESOPE-Equivalent Pulsing Protocols for Calcium Electroporation: An In Vitro Optimization Study on 2 Cancer Cell Models.” Technology in cancer research & treatment vol. 17 (2018): 1533033818788072. doi:10.1177/1533033818788072.

4. De Leonardis, Francesco et al. “In Vitro Effects of Low-energy Ultrasound Treatment on Healthy CD3/CD8+ Lymphocytes, Red blood cells, Acute Myeloid leukemia cells, and Jurkat cell line.” Journal of Cancer vol. 14,7 1088-1106. 24 Apr. 2023, doi:10.7150/jca.83050.
